# Anomaly detection in spatial transcriptomics via spatially localized density comparison

**DOI:** 10.1093/bioinformatics/btaf242

**Published:** 2025-07-15

**Authors:** Gary Hu, Julian Gold, Uthsav Chitra, Sunay Joshi, Benjamin J Raphael

**Affiliations:** Department of Computer Science, Princeton University, Princeton, NJ, 08540, United States; Center for Statistics and Machine Learning, Princeton University, Princeton, NJ, 08540, United States; Eric and Wendy Schmidt Center, Broad Institute of MIT and Harvard, Cambridge, MA, 02142, United States; Department of Applied Mathematics and Computational Sciences, University of Pennsylvania, Philadelphia, PA, 19104, United States; Department of Computer Science, Princeton University, Princeton, NJ, 08540, United States

## Abstract

**Motivation:**

Perturbations in biological tissues—e.g. due to inflammation, disease, or drug treatment—alter the composition of cell types and cell states in the tissue. These alterations are often spatially localized in different regions of a tissue, and can be measured using spatial transcriptomics technologies. However, current methods to analyze differential abundance in cell types or cell states, either do not incorporate spatial information—and thus cannot identify *spatially localized* alterations—or use heuristic and inaccurate approaches.

**Results:**

We introduce *Spatial Anomaly Region Detection in Expression Manifolds* (*Sardine*), a method to estimate spatially localized changes in spatial transcriptomics data obtained from tissue slices from two or more conditions. *Sardine* estimates the probability of a cell state being at the same (relative) spatial location between different conditions using spatially localized density estimation. On simulated data, *Sardine* recapitulates the spatial patterning of expression changes more accurately than existing approaches. On a Visium dataset of the mouse cerebral cortex before and after injury response, as well as on a Visium dataset of a mouse spinal cord undergoing electrotherapy, *Sardine* identifies regions of spatially localized expression changes that are more biologically plausible than alternative approaches.

**Availability and implementation:**

We implement *Sardine* in Python 3, with an open source implementation available at: https://github.com/raphael-group/spatial_anomaly_detection.

## 1 Introduction

A key problem in tissue biology is characterizing how disease, experimental perturbations, drug treatments, and other changes in biological conditions alter the composition of cell types and cell states in a tissue (Levine *et al.* 2015). By comparing transcriptomics data from samples collected under both biological conditions, unique condition-specific gene expression profiles and cell types have been uncovered that elucidate the molecular changes associated with these changes in condition (Sade-Feldman *et al.* 2018, Jin *et al.* 2020).

A common approach to quantify changes in cell state across biological conditions is to measure single-cell RNA sequencing (scRNA-seq) data under each biological condition, and identify cell states that are altered between the different conditions. There are many different methods for solving this problem, each of which makes a different choice on defining a cell state and quantifying the alteration of a cell state. Typically, the cell states are defined in terms of “neighborhoods” of a *k*-nearest neighbor (kNN) graph of the data, while alterations to cell state are defined in terms of differential abundance (Dann *et al.* 2022), kernel density estimates (KDEs) (Burkhardt *et al.* 2021), or regression (Zhao *et al.* 2021). Importantly, Zhao *et al.* (2021) and Landa *et al.* (2020) derive a statistically rigorous formulation of this problem as a “localized two-sample testing” problem. There are multiple computational challenges involved in identifying diferential abundance from by scRNA-seq data, including the high-dimensionality (≈20000 genes) and sparsity of the data (the expression matrix can be 0 in >80% of entries), as well as batch effects between different datasets that are not indicative of real biology (Stuart *et al.* 2019).

Importantly, differences in cell types and cell states between conditions are often *spatially localized* (Peng *et al.* 2024, Zhou *et al.* 2024). scRNA-seq lacks the spatial information to quantify such spatially localized changes, but newer spatial transcriptomics (ST) technologies provide such data. Numerous methods have been introduced to identify spatially varying genes (SVG) (Svensson *et al.* 2018, Chitra *et al.* 2025) and derive spatial domains (Singhal *et al.* 2024, Varrone *et al.* 2024, Chitra *et al.* 2025) from ST data of a single tissue slice, or to aligning ST data from multiple adjacent slices of a tissue (Zeira *et al.* 2022, Clifton *et al.* 2023, Liu *et al.* 2023). However, none of methods quantify changes in the likelihood of observing *cell states* within particular spatial regions of ST data. The two existing computational methods to identify spatially localized differential abundance, STANDS (Xu *et al.* 2024) and Vespucci (Teo *et al.* 2024) rely on heuristics measures to quantify spatially localized changes in expression. Specifically, STANDS (Xu *et al.* 2024) uses a Generative Adversarial Network (GAN)-based approach trained control condition slices and evaluated on the perturbed condition slices, while Vespucci (Teo *et al.* 2024) trains a separate random forest classifier in local spatial windows to classify spots into conditions based on gene expression, with the accuracy of the classifier quantifying the change in expression.

The problem of identifying cell states altered across conditions is closely related to the well-studied *spatial anomaly detection* problem in statistics and machine learning. The standard approach to detect spatial anomalies is the spatial scan statistic (Kulldorff 1997, Glaz *et al.* 2001, Chitra *et al.* 2021), where a spatial anomaly is computed as a spatial region that maximizes the value of a test statistic, e.g. likelihood ratio test. With few exceptions (Frévent *et al.* 2023), spatial scan statistics are typically designed for point processes and have not been successfully applied to high dimensional or sparse data as observed in ST.

**Figure 1.**
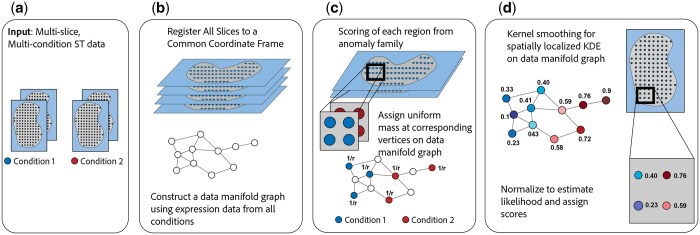
Overview of *Sardine*. (a) The inputs are spatial transcriptomics data from one or more slices from two or more conditions. (b) Slides are aligned to a common coordinate system and a data manifold graph is constructed from the expression data from all slices in all conditions. (c) Each region from the anomaly family is evaluated. Uniform probability mass is placed on each vertex of the data manifold corresponding to a location in the region. (d) Graph smoothing of initial probability mass gives density estimates for the spatial region on the entire manifold graph. Comparison of density estimates between conditions defines regions of interest.

In this work, we formulate the *spatially localized differential abundance region problem* (SDARP) of identifying spatial regions that have a differential abundance of cell states between conditions, as defined by densities on a cell state manifold. The SDARP is an extension of the differential abundance problem (Burkhardt *et al.* 2021, Zhao *et al.* 2021, Dann *et al.* 2022) which defines regions on a cell state manifold that differ in density between two conditions. We introduce *Spatial Anomaly Region Detection in Expression Manifolds* (*Sardine*) (Fig. 1) which solves the SDARP by combining two components: (i) a spatial *anomaly family* R (Chitra *et al.* 2021), defining the spatial regions of interest; and (ii) a plug-in density estimator for the density f(x) given samples {x} drawn from f.

We evaluate *Sardine* on multiple simulated datasets, outperforming existing approaches in recapitulating changes in gene expression distributions in space. On a Visium dataset of mouse cortex injury response (Koupourtidou *et al.* 2024), *Sardine* recovers the expected wound location, and finds a perturbation region that is more biologically plausible then competing methods. Finally, on a Visium experiment conducted on mouse spinal cords undergoing different therapies (Squair *et al.* 2021), *Sardine* recovers meaningful spatial regions which correlate more strongly with the expression of condition-associated genes than competing methods.

## 2 Materials and methods

### 2.1 Spatially localized differential abundance problem

We formulate the problem of anomaly detection on spatial transcriptomics data as comparing *spatially localized* probability densities of gene expression between each condition. Under the manifold hypothesis, high-dimensional gene expression data from two biological conditions ${\left\{x_{i}\right\}}_{i=1}^{n}\cup {\left\{x_{i}^{\prime }\right\}}_{i=1}^{n^{\prime }}\subset R^{g}$ are sampled from densities $f_{0},f_{1}$ supported on a lower dimensional manifold $M\subset R^{g}$. Within a subset S⊂M, the *abundance* of a condition is roughly the number of samples in *S* from this condition. *Differential abundance* within *S* compares the proportion of samples in *S* from each condition, and is thus related to the density difference $f_{1}-f_{0}$. In the single-cell context, the *anomalous region* is the subset of M where $f_{1}>f_{0}$, and any score of differential abundance can be used to estimate which of the data lie in this anomalous region.

Before formulating the spatially localized anomaly detection problem for spatial transcriptomics data, we first formulate anomaly detection in the nonspatial (single-cell) setting. The gene expression data of any single-cell or ST experiment is represented as a matrix $X\in R^{n\times g}$, where *n* is the number of spots or cells depending on technology, and *g* is the number of genes sequenced in each spot. For ease of notation we consider the two-condition setting (e.g. healthy versus diseased) in the problem formulation, but note that *Sardine* is easily applied on multiple conditions. In this two-condition setting, we are given a matrix for each condition: $X,X^{\prime }$, where the rows of each matrix, i.e. ${\left\{x_{i}\right\}}_{i=1}^{n},{\left\{x_{i}^{\prime }\right\}}_{i=1}^{n^{\prime }}$, are assumed to be drawn from $f_{0}$ and $f_{1}$.

As in existing literature (Kim *et al.* 2019, Landa *et al.* 2020, Burkhardt *et al.* 2021) the main quantity of interest is the relative likelihood of a condition label Z={0,1} for a given observation of a cell’s gene expression, which is written in terms of the densities as:


(1) $$\begin{array}{c} P(Z=1|x)=\frac{f_{1}(x)}{f_{1}(x)+f_{0}(x)}\\ P(Z=0|x)=\frac{f_{0}(x)}{f_{1}(x)+f_{0}(x)} \end{array}$$

Formally, we define the *anomalous set* A={x∈M:P(Z=1|x)>P(Z=0|x)} in terms of (1), but this region can equivalently be defined as $A=\{x\in M:f_{1}(x)>f_{0}(x)\}$, the region(s) of the expression manifold where the density of the perturbed condition is larger than the density of the healthy condition. We note briefly that it is arbitrary which condition is assigned to condition labels 0 and 1, and one can flip the condition labels such that the perturbed condition corresponds to 0 to perform comparisons in the opposite direction. At a high level, methods to determine A typically aim to return the subset of the observed datapoints within A. The general Differential Abundance Problem is:

Problem 1 ***Differential Abundance Problem (DAP):*** *Let* $f_{0},f_{1}$  *be Lipschitz probability densities supported on manifold* $M\subset R^{d}$*, and let* $X,X^{\prime }$  *be expression matrices whose rows are i.i.d samples from* $f_{0},f_{1}$*, respectively. Given* $X,X^{\prime }$*, find the intersection* $(X\cup X^{\prime })\cap A$  *of the data and the anomalous region.*

If density functions $f_{0},f_{1}$ were given or were able to be accurately estimated, then determining A, and by extension solving DAP, would be trivial. In practice, estimating these density functions directly is an extremely challenging problem, and is intimately related to density difference estimation (Sugiyama *et al.* 2013) and local two-sample testing (Kim *et al.* 2019, Landa *et al.* 2020, Kim *et al.* 2021). In particular, (Landa *et al.* 2020) developed a robust statistical framework for local two-sample testing using random walk neighborhoods intrinsic to the data, especially suited to high-dimensional data under the manifold hypothesis. We outline the local two-sample testing framework in Supplementary Section S4.1.

A number of recent works (Burkhardt *et al.* 2021, Skinnider *et al.* 2021, Zhao *et al.* 2021, Dann *et al.* 2022) estimate a discrepancy between condition densities $f_{0}$ and $f_{1}$, with (Burkhardt *et al.* 2021, Dann *et al.* 2022) in the spirit of general scan statistics (Landa *et al.* 2020), while classifier-based (Skinnider *et al.* 2021, Zhao *et al.* 2021) are more related to the framework of (Kim *et al.* 2021). As the fundamental comparison within the DAP is the comparison of $f_{1}(x)>f_{0}(x)$, the works (Burkhardt *et al.* 2021, Zhao *et al.* 2021, Dann *et al.* 2022) each yield solutions to the DAP. We include a comparison of these previous works in Supplementary Section S4.2.

Despite these advancements in solving DAP for scRNA-seq, naively solving the DAP problem on the expression data of ST is insufficient to accurately detect and quantify the anomalies in spatial data. One must also consider changes in the spatial organization of expression within the tissue, which can be spatially coherent and localized to subregions of the tissue. An analysis restricted to the expression components of the data could underrepresent or obscure any spatial organization. As an extreme hypothetical, permuting all spatial positions of all spots, while leaving expression unchanged, destroys the ability to recognize spatially coherent anomalies. However, the aforementioned single-cell methods would not change their analysis.

For the spatial anomaly problem, we represent spatial transcriptomics datasets of multiple conditions as two tuples: $Y=(X,S),Y^{\prime }=(X^{\prime },S^{\prime })$, where $S,S^{\prime }\in R^{n\times 2}$ represent the spatial coordinates. We assume that the coordinate data for both conditions have already been aligned onto the same common coordinate system. Alignment between slices is a well tackled problem, with many recently developed computational methods (Zeira *et al.* 2022, Clifton *et al.* 2023, Liu *et al.* 2023, Hu *et al.* 2024).

In this setting, the joint spatial and transcriptomic features y=(x,s) for each spot are drawn from joint densities $h_{0},h_{1}$ on $R^{g+2}$, with one density per condition. We continue to use $f_{0}$ and $f_{1}$ for the now marginal densities of each condition in expression space, and we additionally write $\rho_{0}$ and $\rho_{1}$ for the spatial marginal densities of each condition. Let $f_{0}(\cdot |s)$ and $f_{1}(\cdot |s)$ denote densities of each condition in expression space, conditional on the value of spatial coordinate s. The posterior probability of class variable *Z* given x and s is, for i=0,1:


(2) $$P(Z=i|x,s)=\frac{h_{i}(x,s)}{h_{1}(x,s)+h_{0}(x,s)},$$

which we may equivalently express as:


(3) $$P(Z=i|x,s)=\frac{f_{i}(x|s)\rho_{i}(s)}{f_{1}(x|s)\rho_{1}(s)+f_{0}(x|s)\rho_{0}(s)},$$

We now introduce a simplifying assumption, namely that the two conditions are *spatially registered*. This is defined to mean that $\rho_{0}$ and $\rho_{1}$ are identical, and that spatial data $S={\left\{s_{i}\right\}}_{i=1}^{n},S^{\prime }={\left\{s_{i}^{\prime }\right\}}_{i=1}^{n^{\prime }}$ can be treated as lying in a common coordinate system. When Y and $Y^{\prime }$ are spatially registered, the above posterior probabilities simplify into:


(4) $$\begin{array}{c} P(Z=1|x,s)=\frac{f_{1}(x|s)}{f_{1}(x|s)+f_{0}(x|s)}\\ P(Z=0|x,s)=\frac{f_{0}(x|s)}{f_{1}(x|s)+f_{0}(x|s)} \end{array}$$

Define the *localized anomalous set* $A_{S}=\{(x,s)\in M\times R^{2}:P(Z=1|x,s)>P(Z=0|x,s)\}$. The Spatially localized Differential Abundance Problem is then a natural extension of the DAP:

Problem 2 ***Spatially localized Differential Abundance Problem (SDAP):*** *Let* Y=(X,S)  *and* $Y^{\prime }=(X^{\prime },S^{\prime })$  *have expression matrices* $X,X^{\prime }$  *as in Problem 1. Suppose also that* S  *and* $S^{\prime }$  *are spatially registered. Given* $Y,Y^{\prime }$*, find* $(Y\cup Y^{\prime })\cap A_{S}$.

The estimation of $f_{1}(\cdot |s),f_{0}(\cdot |s)$ from ST data is even more challenging than inferring $f_{0},f_{1}$ from expression, as one must estimate a density over the gene expression manifold given at most a couple of data points at each spatial location s. Furthermore, existing methods for density estimation rely on quantifying distances between data points, but in general, there is no canonical distance between two ST spots that does not make some heuristic decision as to the relative scaling of gene expression and physical distance, hindering interpretability.

We propose to use averaging associated to more classical spatial scan statistics Kulldorff (1997) to make the density comparison between conditions feasible on ST data. Given spatial region $R\subset R^{2}$ within the assumed common spatial coordinate system, let $f_{0}(x|R),f_{1}(x|R)$, denote the gene expression densities of each condition, with densities now conditioned on the event {s∈R}. Computing $f_{0}(x|R),f_{1}(x|R)$ is more tractable as observations are binned via spatial closeness. Moreover, under the assumption that the conditional densities $f_{k}(\cdot |s)$ vary continuously as a function of s, one should have $f_{k}(\cdot |s)\approx f_{k}(\cdot |R)$ for R∋s of sufficiently small diameter, for k=0,1. It is in this sense that Problem 3 below is an approximation of Problem 2. To formalize this approximation, let $R={\left\{R_{s}\right\}}_{s\in R^{2}}$ be a family of neighborhoods for each point $s\in R^{2}$, which we refer to as a *spatial anomaly family* (Chitra *et al.* 2021). We assume that each of these regions is compact (closed and bounded) and simply connected, with $s\in R_{s}$. Define:


(5) $$\bar{P}(Z=1|x,s)=\frac{f_{1}(x|R_{s})}{f_{1}(x|R_{s})+f_{0}(x|R_{s})},$$

(6) $$\bar{P}(Z=0|x,s)=\frac{f_{0}(x|R_{s})}{f_{1}(x|R_{s})+f_{0}(x|R_{s})}.$$

Problem 3 ***Spatially localized Differential Abundance Region Problem (SDARP):*** *Let* Y=(X,S)  *and* $Y^{\prime }=(X^{\prime },S^{\prime })$  *have expression matrices* $X,X^{\prime }$  *as in Problem 1. Given a spatial window* $R_{s}$  *for each* $(x,s)\in Y\cup Y^{\prime }$*, find* $\bar{P}(Z=1|x,s)$  *for all* $(x,s)\in Y\cup Y^{\prime }$.

As with Problem 1 and Problem 2, there is a corresponding anomalous set for Problem 3. Define the *region-localized anomalous set* $A_{S}(R)=\{(x,s)\in M\times R^{2}:\bar{P}(Z=1|x,s)>\bar{P}(Z=0|x,s)\}$. We note that to solve Problem 3, one only needs R to be a family of spatial neighborhoods indexed by each s among the spatial data $S\cup S^{\prime }$. We emphasize that the SDARP problem is fundamentally interested in finding anomalous regions, rather than specific spots or cells. Moreover, any solution to Problem 3 yields the intersection of the data $Y\cup Y^{\prime }$ and the anomalous set $A_{S}(R)$.

### 2.2 Solving SDARP with *Sardine*

*Sardine* solves the SDARP problem by combining two components: (i) a spatial *anomaly family* R (Chitra *et al.* 2021, Reyna *et al.* 2021, Chitra *et al.* 2022), defining the spatial regions of interest; and (ii) a plug-in estimator ${\tilde{f}}_{0}(x)$ and ${\tilde{f}}_{1}(x)$ of the values $f_{0}(x)$ and $f_{1}(x)$ at each sample $x\in X\cup X^{\prime }$. From these components *Sardine* estimates class posteriors, Equations (5) and (6), by constructing an estimator for the main quantity $\tilde{P}(Z=1|x,s)$

For the first component, we take inspiration from classical scan statistics (Kulldorff 1997) and define $R_{i}$ as a spatial ball around each spot with equally many samples from each condition. We take the closest *r* spots from each condition to each spot *i*, where *r* is a user defined hyperparameter. Importantly, the spatial registration of the datasets ensures that *r* determines largely overlapping neighborhoods of similar diameters across conditions.

For the second component, we require any plug-in estimator of the values $\left\{f(x_{i})\right\}$ given samples $\left\{x_{i}\right\}$, where *f* is the probability density that $\left\{x_{i}\right\}$ are drawn from. However, because we only require density evaluations at the observed data points, and not interpolation to unseen points, one convenient approach is to compute density estimates directly on the support of a similarity graph of the data (Mack and Rosenblatt 1979, Von Luxburg and Alamgir 2013, Senelle 2014, Burkhardt *et al.* 2021). Such an approach has already been successfully applied on scRNA-seq in MELD (Burkhardt *et al.* 2021), with nice theoretical properties as an interpretation of kernel density estimation (KDE) to the discrete graph space while also being extremely fast (density estimates at observed samples takes seconds for thousands of observations). We briefly review the main idea of this process.

Let G=(V,E) be a similarity graph formed from the expression data $X,X^{\prime }$, such as one using *k*-nearest neighbors. We call G the *data manifold* due to distances in this graph approaching distances in manifold M as the number of samples approaches the limit (Tan and Cheng 2024).

For a given condition of interest, let $m\in R^{n+n^{\prime }}$ be the uniform probability vector over this condition. Following Burkhardt *et al.* (2021), density values $d^{⋆}\in R^{n+n^{\prime }}$ for this condition restricted to the data are estimated by the following optimization:


(7) $$d^{⋆}=\text{arg}\;{\text{min}}_{d}||m-d{||}_{2}^{2}+\beta d^{T}\mathrm{Jd},$$

where


(8) $$J=\text{exp}(\beta {\left(L/\lambda_{\max}-\alpha I\right)}^{\rho })-I.$$

Above, L the graph Laplacian of G, assumed undirected or symmetrized so that L is itself symmetric. Parameter α in (8) modulates the strength of the diagonal elements of the Laplacian, while β in (7) controls the strength of the Laplacian regularization. The optimization in (7) averages condition indicator vector m along the entire data manifold to yield density estimate $d^{⋆}$ for the given condition. This process is repeated for each condition to compute density estimates ${\tilde{f}}_{0}(x_{i}),{\tilde{f}}_{1}(x_{i})$ for $x_{i}\in X\cup X^{\prime }$.

To do so, we first utilize the plug-in density estimator to compute $f_{0}(x|R_{i}),f_{1}(x|R_{i})$ for a given $R_{i}$. The approach of (Burkhardt *et al.* 2021) is easily extended to accommodate this, as replacing the uniform vector m on the condition of interest with a spatially localized uniform vector $m_{R_{i}}$ supported on points in the same condition that additionally lie in $R_{i}$. The density estimate then proceeds through Equation (7) as in the original work, resulting in pointwise estimates ${\tilde{f}}_{0}(x|R_{i})$, ${\tilde{f}}_{1}(x|R_{i})$.

Following (Burkhardt *et al.* 2021), these density estimates are normalized to get the relative likelihood estimate ${\tilde{Q}}_{i}=\frac{{\tilde{f}}_{1}(x_{i}|R_{i})}{{\tilde{f}}_{0}(x_{i}|R_{i})+{\tilde{f}}_{1}(x|R_{i})}$. Similar to the weighted averaging of pointwise density discrepancies in (Landa *et al.* 2020) when finding regions of differential abundance on the cell state manifold with statistical guarantees, we argue that a more robust estimation given the combination of the spatial anomaly family $R_{i}$ and the plug in density estimator is the average likelihood of the perturbed spots in $R_{i}$: $\frac{1}{r}\sum_{j\in R_{i}\cap S^{\prime }}{\tilde{Q}}_{j}$. By using the density estimates from both conditions, if the densities ${\tilde{f}}_{0}(x|R_{i})$ and ${\tilde{f}}_{1}(x|R_{i})$ are similar, the resulting likelihoods will be 0.5 for the perturbed samples.

In a similar vein to how differential abundance approaches (Landa *et al.* 2020, Zhao *et al.* 2021) rely on local averaging in expression space, here the locality is defined in physical space. However, we deviate from scRNA-seq differential abundance methods as they focus on finding manifold regions where one condition is more abundant than the other. Due to our construction of the spatial anomaly family R, each $R_{i}$ has the same number of samples from both conditions. Thus, we use the evaluation of the perturbed spots when estimating the likelihood of the perturbed condition in Equation (5). We synonymously call our final likelihood estimate $\frac{1}{r}\sum_{j\in R_{i}\cap S^{\prime }}{\tilde{Q}}_{j}$ the *anomaly score* for spot $s_{i}$, and in our results we label each spot by this anomaly score.

## 3 Results

### 3.1 Analysis on simulated spatial data

We evaluated *Sardine* on multiple simulated ST datasets. We compared *Sardine* against two methods to detect anomalies from single-cell and ST data: MELD (Burkhardt *et al.* 2021), the state-of-the-art method for detecting anomalies in (non-spatial) single-cell data; and Vespucci (Teo *et al.* 2024), a recent method for detecting anomalies from ST data. We do not compare against STANDS (Xu *et al.* 2024), another recent method for anomaly detection method in ST data, as it requires histology images.

**Figure 2.**
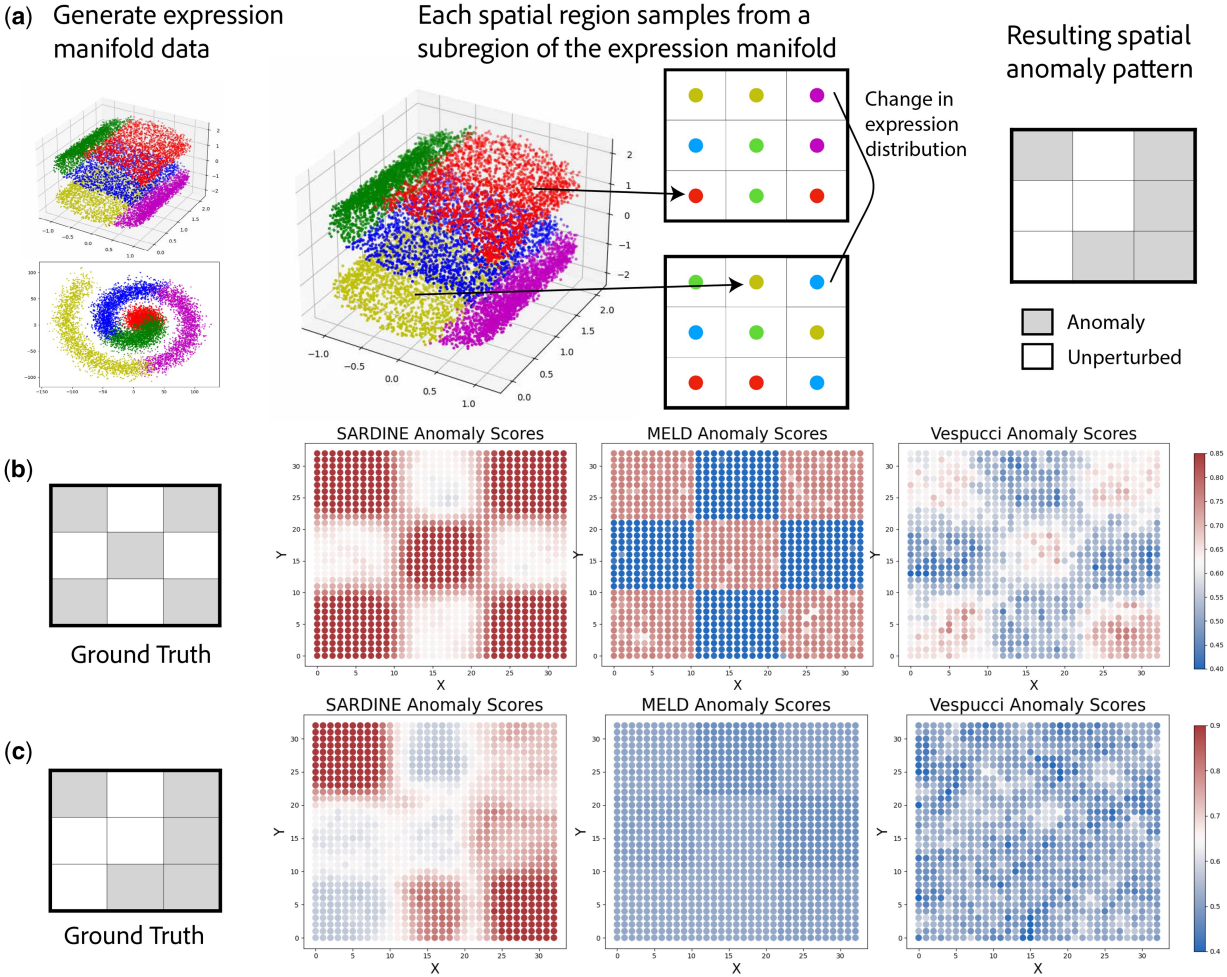
Benchmarking *Sardine* on simulated data. (a) We create synthetic features that follow the manifold hypothesis, where the high dimensional (d=100) feature vectors originate from a low dimensional (d=2,3) manifold. We simulate ST data by creating a 2-D coordinate grid and assign a subregion of the expression space to each spatial subregion. This procedure is repeated to create 2 artificial slices corresponding to different conditions. About half of the corresponding spots between conditions will be drawn from the same region of the manifold, and half will be drawn from distinct regions. (b) Results for *Sardine*, MELD, and Vespucci, where gene expression follows one of two multivariate normal distributions in the anomaly and unperturbed subregoins. (c) Results for *Sardine*, MELD, and Vespucci, where gene expression is sampled from the S-curve manifold.

For each simulation experiment, we simulate two ST datasets (X,S) and $\left(X^{\prime },S\right)$. The spatial coordinates S form a N×N square grid (N=33) and we define an anomalous region A according to one of two different spatial patterns:

- *Checkerboard*: we partition the spatial locations into 9 smaller N/3×N/3 squares $Q_{1},\ldots ,Q_{9}$. The anomalous region A is the union of a subset of the nine squares (5 in Fig. 2a).
- *Spiral*: The anomalous region A occupies a spiral shape in $R^{2}$ (Supplementary Section S1.4).

The distribution of each sample $x_{i},x_{i}^{\prime }$, i.e. the rows of the two respective gene expression matrices $X,X^{\prime }\in R^{N\times G}$, depends on whether the spatial location i∈{1,…,N} is in the anomaly A: if i∈A, then both samples $x_{i},x_{i}^{\prime }∼P_{i}$ are sampled from the same distribution $P_{i}$; otherwise, the first sample $x_{i}∼Q_{i}$ is drawn from $Q_{i}$ and the second sample $x_{i}^{\prime }∼Q_{i}^{\prime }$ is drawn from $Q_{i}^{\prime }$. For the multivariate normal simulations, $P=Q,P,Q^{\prime }$ are both multivariate normal distributions of dimension (d=100) with different mean vectors. For the S-curve and spiral manifold distributions, we simulated samples according to a low dimensional manifold structure (Fig. 2a).$P,Q,Q^{\prime }$ are uniform distributions over the finite set of samples within a certain partition of the manifold. This simulation approximates sampling from a continuous manifold while maintaining interpretability of the experiment. These partitions are visualized in (Fig. 2a), with some addition info in Supplementary Section S1.

We use one set of hyperparameters for *Sardine* (β=10,knn=8); based on robust performance across simulation setups while performing hyperparameter sweeps (Supplementary Section S1). We set the spatial window size for both *Sardine* and Vespucci to be equal (r=30).

Since all methods report anomaly *scores* $z_{i}$ for each spatial location, we evaluated each method in two ways: we compute the area under the receiver operating characteristic curve (AUROC) of the anomaly scores $z_{i}$ compared to the true anomaly labels $\ell_{i}=1_{\left\{i\in A\right\}}$; and we compute the Mean Squared Error $\sum_{i=1}^{N}(d(z_{i})-\ell_{i}{)}^{2}$, where we define d(zi)=0.5·1{i∈A}+1·1{i∈A}, i.e. $d(z_{i})$ is 0.5 for spatial locations not in the anomaly and $d(z_{i})=1$ for locations in the anomaly. The idealized scores matches the the interpretation of the anomaly scores of each method. This metric quantifies not just whether these methods can identify anomalous spots from non-anomalous, but also the separation in the anomaly scores themselves.

We find that *Sardine* consistently outperforms MELD and Vespucci (Fig. 2b and c). The anomaly scores of *Sardine* have lower MSE on all simulated expression distributions (Table 1), and *Sardine* has the highest AUROC in all settings except for one. On the checkerboard spatial anomaly with multivariate normal distributed features, MELD reported anomaly scores in non-anomalous spots as lower than the expected 0.5 (Fig. 2b), which is incorrect, as it compares the features at each spot to the global density values rather than spatially local ones.

**Table 1.** Quantitative metrics of *Sardine*, MELD, and Vespucci on four simulated datasets. Bolded numbers indicate the best observed values for each metric and dataset.

Expression distribution	Spatial anomaly pattern	*Sardine* MSE	MELD MSE	Vespucci MSE	*Sardine* ROC AUC	MELD ROC AUC	Vespucci ROC AUC
Multivariate normal	Checkerboard	**0.022**	0.039	0.081	1.0	1.0	0.953
S-curve manifold	Checkerboard	**0.039**	0.138	0.138	**0.984**	0.485	0.456
Spiral manifold	Checkerboard	**0.042**	0.082	0.060	**0.985**	0.672	0.819
Multivariate normal	Spiral shaped	**0.050**	0.061	0.066	0.810	**0.817**	0.602

Sardine requires O(n) density evaluations, where *n* is the total number of spots. With our current implementation, Sardine takes ≈30 seconds to run on each simulated dataset. This is slightly slower than just using MELD (≈1 second), but much faster than Vespucci (>20 min).

### 3.2 Analysis of the post-injury mouse cerebral cortex

**Figure 3.**
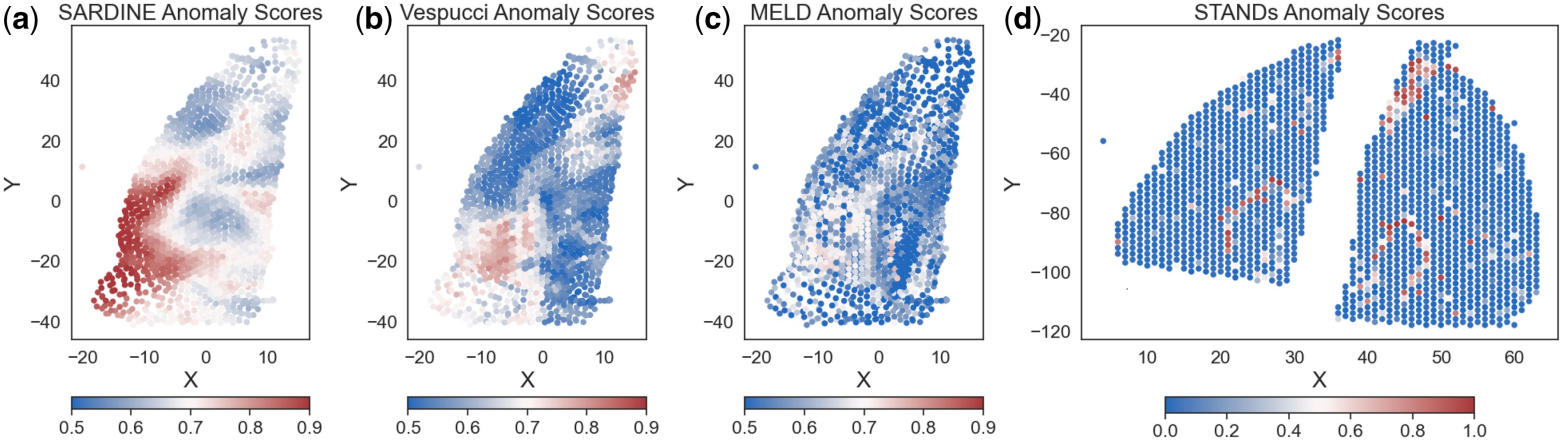
Mouse cortex stab results. Anomaly scores for (a) *Sardine*, (b) Vespucci, (c) MELD, and (d) STANDS. Each method returns an anomaly score between 0 and 1 for each spot, representing how anomalous that spot is, with the first 3 methods reporting 0.5 as equally likely to be anomalous or not. The stab wound site is located in the outer layer of the cortex. On the registered slices, it is located on the lower left side. The spatial coordinates of STANDS are the original two replicates, due to the correspondence with histology images. The aligned coordinates have these two slices mapped onto the same common coordinate space.

We next applied *Sardine*, Vespucci, MELD, and STANDS on a real Visium dataset of a healthy and post stab injury mouse cerebral cortex (Koupourtidou *et al.* 2024). Each condition had two replicates, giving four total replicates to consider. We aligned all replicates into a common coordinate system (CCS) using STalign (Clifton *et al.* 2023), a state-of-the-art spatial alignment method. As STANDS makes use of histology images, we did not perform alignment on the coordinate information when running that method, which is reflected in the visualization of the results in Fig. 3.

The injury site consists of a stab wound into the cortical region, which in the CCS corresponds to the lower left part of the tissue. We include a diagram from the original authors of the experimental study (Koupourtidou *et al.* 2024) in Supplement Section S2.1. *Sardine*, Vespucci, and to a lesser extent MELD identify this lower left region as anomalous, which serves as a useful ground truth. Visually, *Sardine* identifies an anomalous region that more closely follows the geometry of the layered structure of the cerebral cortex beyond the stab wound, matching the original assumptions of (Koupourtidou *et al.* 2024). The cerebral cortex region is a layer on the left side of the tissue on the CCS. STANDS completely misses the injury site as an anomalous region. Instead, it prioritizes the hippocampal formation, a U-shaped structure responsible for key brain functions, but not the most transcriptionally perturbed. Both *Sardine* and Vespucci report regions that are spatially coherent, whereas MELD and STANDS do not, indicated by computing Moran’s I values for each of the methods’ anomaly scores (Table 2).

**Table 2.** Moran’s I values for the anomaly scores of each method on the mouse cortex stab wound dataset. Bolded numbers indicate the best observed value.

Anomaly detection method	Moran’s I
*Sardine*	**0.883**
Vespucci	0.825
MELD	0.259
STANDS	0.147

**Figure 4.**
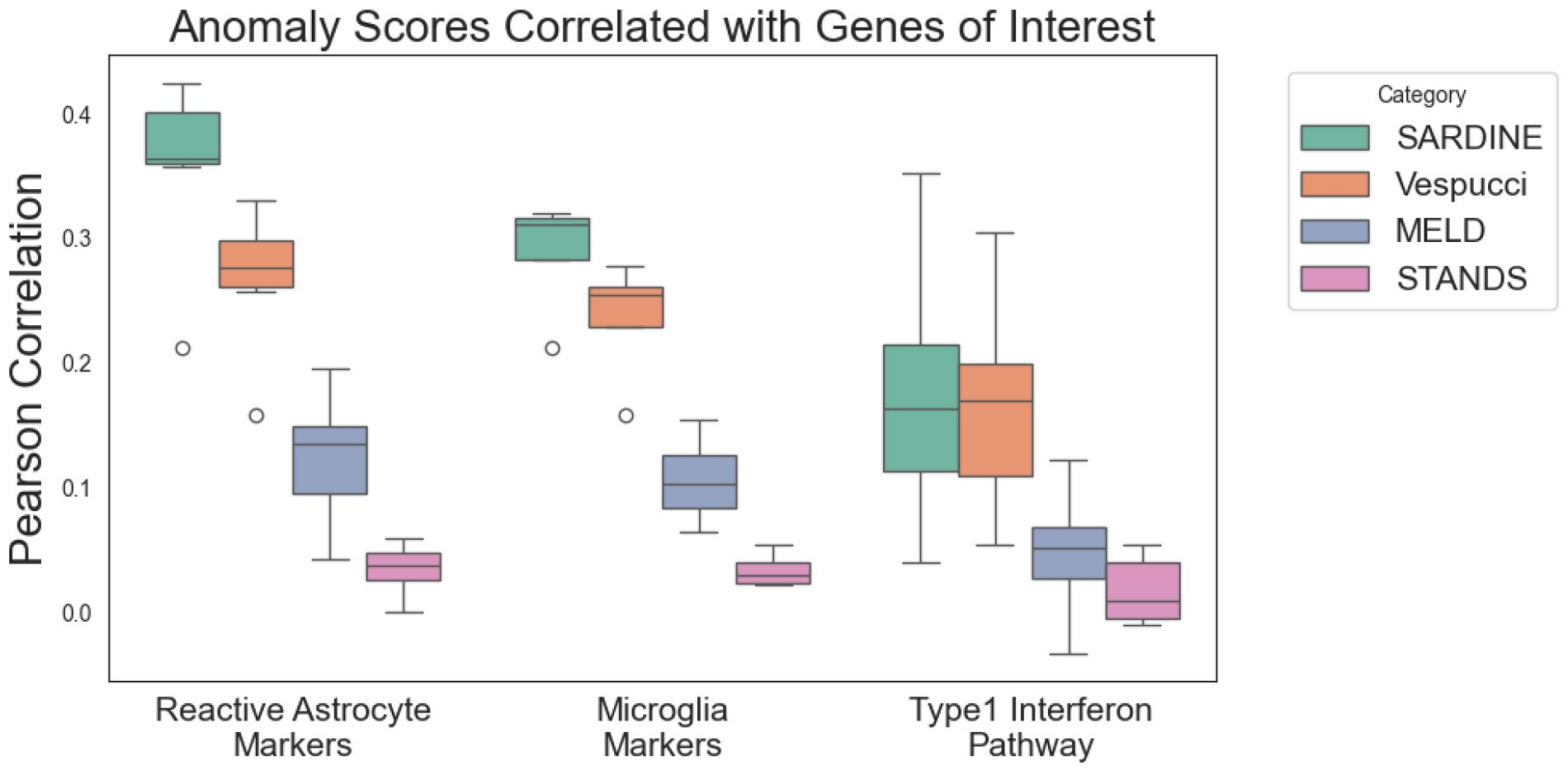
Mouse cortex stab correlations. Points in the boxplot are individual genes, grouped together into sets based on what the gene is a marker for.

We next tested whether the anomaly scores of *Sardine* better corresponded to expected biological shifts in the expression of certain genes. The original study by (Koupourtidou *et al.* 2024) mentions certain marker genes as notable. Among these include marker genes for reactive astrocytes, which are a cell type that specifically appears in response to injury, the microglia, which have also been reported to be positively associated with injury response. and genes associated with the Type 1 Interferon pathway, which they report as being impacted by the injury. We list the specific genes in Supplementary Section S2.7. For all of these gene sets, we compute the Pearson correlation of each gene in the gene set within the injured replicates to the reported anomaly scores. *Sardine* reported the highest correlations in both the Reactive Astrocyte Markers and the Microglia markers, while reporting slightly smaller correlations on the Type 1 Interferon pathway, though the Type 1 Interferon pathway was the lowest correlating gene set among the list (Fig. 4).

Finally, we determined whether the anomaly scores reported by *Sardine* correlated with gene sets beyond those originally reported. To answer this, we ran a Gene Set Enrichment Analysis (GSEA) (Zhang *et al.* 2005, Elizarraras *et al.* 2024), where we report a ranked list of all genes by Pearson correlation of their expression to the anomaly score of each method on a per spot basis. The number of significant (FDR <0.05) gene sets, with represent biological phenotypes, for each method is shown in Table 3 and Supplementary Section S2.8. *Sardine* reported an order of magnitude more significant hits than other methods. Because the cortex is normally composed of very spatially coherent regions, and similar cell states in similar locations should be perturbed in similar ways, this suggests that the anomalous regions reported by *Sardine* are more plausible than those found by other methods. Furthermore, many of the significant gene sets are strongly associated with cortex injury response, including abnormal acute inflammation (FDR=2.1e−4), abnormal innate immunity (FDR=4.2e−5), and abnormal dendritic cell physiology (FDR=<2.2e−16), all of which are missing among the significant gene sets reported by other methods.

**Table 3.** Mouse cortex stab wound injury.^a^

Method name	No. of significant gene sets from GSEA
*Sardine*	650
Vespucci	26
Meld	0
STANDS	1

a Number of significant (FDR <0.05) reported gene sets based on GSEA analysis of each method’s anomaly scores.

### 3.3 Analysis of the mouse lumbar spinal cord after neuro-rehabilitation

**Figure 5.**
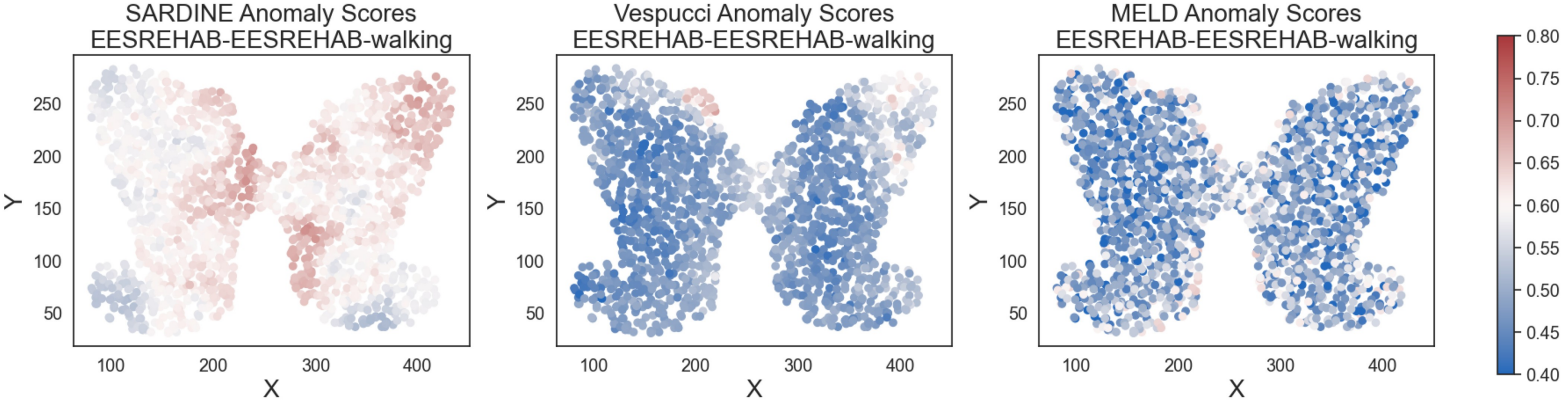
Spinal cord injury analysis. (a) The anomaly scores for *Sardine*, Vespucci, and MELD. *Sardine* returns anomaly scores which highly distinct and spatially coherent regions of the tissue.

We also ran *Sardine* on a multi-condition spinal cord dataset originally published by (Kathe *et al.* 2022). In this study, mice were subjected to a severe spinal cord injury (SCI), followed by various regimes of epidural electrical stimulation (EES) therapy. There are four different conditions (SCI, EESREHAB, EESREHAB-walking, and SCI-EES-walking), which yields six different pairwise comparisons between conditions. We applied *Sardine*, Vespucci (Teo *et al.* 2024), and MELD (Burkhardt *et al.* 2021) to all six pairs of conditions. We visualize one such pairwise comparison in (Fig. 5). STANDS was not run as the available data was pre-aligned to be on a common coordinate system, thus there was not an established mapping between the numerical coordinates provided and the histology images. Following (Kathe *et al.* 2022, Teo *et al.* 2024), we restrict analysis to the grey matter portion of the spinal cord.

Of the three methods tested, *Sardine* recovered the most spatially coherent anomalous regions (Table 4 and visualized in Supplementary Section S3.5) on all pairs of conditions. Unlike the mouse cortex injury dataset, which had a known injury location, for this data nothing about the location of the anomalous region is known. However, we still expect the anomalies to be spatially coherent, as we expect cells close in both expression and space should exhibit similar perturbed expression profiles after injury. Thus the lower coherence of the competing methods’ anomaly scores suggests that they may not be as plausible as the anomalies reported by *Sardine*.

**Table 4.** Moran’s I values for the anomaly scores of each method on the mouse cortex stab wound dataset, across each of the (6 choose 2) pairs of conditions. Bolded numbers indicate the best observed value for each condition.

Cond. 1	Cond. 2	*Sardine* Moran’s I	Vespucci Moran’s I	MELD Moran’s I
SCI	EES REHAB	**0.913**	0.884	0.013
SCI	EES REHAB-walking	**0.890**	0.844	0.0450
SCI	SCI-EES-walking	**0.891**	0.838	0.006
EES REHAB	EES REHAB-walking	**0.800**	0.699	0.092
EES REHAB	SCI-EES-walking	**0.824**	0.717	0.072
EES REHAB-walking	SCI-EES-walking	**0.715**	0.544	0.101

To further determine the plausibility of the anomalous regions, we repeated gene set enrichment analysis (GSEA) (Elizarraras *et al.* 2024, Zhang *et al.* 2005) on the genes ranked by Pearson correlation of their expression to the anomaly scores on a per spot basis. We focused on the comparison between the EESREHAB, and EESREHAB-walking conditions. The resulting gene list reported many significant (FDR <0.05) gene sets that correspond with spinal cord injury. These include impaired synaptic plasticity (FDR =0.007), abnormal excitatory postsynaptic currents (FDR =0.006), and many other potentially related terms. We repeated this analysis for the anomaly scores of MELD and Vespucci, but found almost no significant gene sets (Table 5 and Supplementary Section S3.6). Given the larger numbers of both spinal cord injury-related and general gene sets that significantly correlate with *Sardine*, we reason that the anomalous regions of *Sardine* correspond better to the underlying biological change.

**Table 5.** Significant (FDR <0.05) gene sets reported by GSEA using the anomaly scores reported by each method for the spinal cord injury dataset.

Method	No. of significant gene sets from GSEA
*Sardine*	88
Vespucci	4
Meld	0

Finally, *Sardine* provides an interpretable manner to analyze >2 conditions simultaneously. Given averaged density estimates for each condition, we compute a likelihood over all conditions by normalizing density estimates across those from all conditions. We visualize these in Supplementary Section S4.7. These likelihoods are spatially coherent, with Moran’s I values (0.910,0.845,0.841,0.862) for SCI, EESREHAB, EESREHAB-Walking, and SCI-EES-walking, respectively. *Sardine* is more interpretable in multi-condition analysis, where the reported values are a function of the individual conditional densities. Because Vespucci and STANDS are built on binary classifiers, one would have to combine data from multiple conditions, hindering interpretability.

## 4 Discussion

Comparing spatial transcriptomics data across conditions is an increasingly common task. *Sardine* provides a rigorous approach to identify spatially localized changes in abundances of cell types/states. On both simulated and experimental data, *Sardine* identifies spatial anomalies that are more accurate or more biologically plausible than existing methods.

There are several directions for future work. First, the SDARP problem formulation is agnostic to the spatial anomaly family as well as the density estimator. Our formulation thus could easily incorporate alternative constructions of the spatial anomaly family R and different density estimators of $f_{0}(x_{i}|R_{i}),f_{1}(x_{i}|R_{i})$. Previous work across different fields of computational biology and classical statistics emphasizes the possibilities of different constructions of each of these components, which we hope to explore in future work.

Additionally, *Sardine* requires slices registered to a common coordinate system. We do not view this a major limitation as (i) the problem of aligning two or more slices has several existing solutions (Hu *et al.* 2024), and (ii) when slices have different tissue architectures it typically entails stronger assumptions to avoid an undetermined task in determining the mapping. However, many alignment methods assume a correspondence in expression space between mapped spots to improve the alignment, an assumption that may be too strong for slices from different conditions. Combining alignment and spatial anomaly detection may improve the sensitivity and specificity of both approaches.

Another interesting direction is to apply our method to data of different resolutions and modalities. The underlying assumption of our method relies on nearest neighbor graph distances as an approximation for distances on the manifold. The use of such graph is ubiquitous even across different technologies, thus we expect our method to perform well in a wide variety of settings. However, a finer- grained resolution may entail more thoughtful parameter selection or the need to consider spatial anomaly families beyond the spatially nearest.

## Supplementary Material

btaf242_Supplementary_Data

## Data Availability

The mouse cortex stab wound dataset was downloaded from GSE226211, and the spinal cord injury data was downloaded from GSE184369. Code to run *Sardine* is at: https://github.com/raphael-group/spatial_anomaly_detection.
