# Supplementary material for: Anomaly detection in spatial transcriptomics via spatially localized density comparison
Source: Bioinformatics. 2025 Jul 15;41(Suppl 1):i493–501. doi: 10.1093/bioinformatics/btaf242 (PMC12261494; doi:10.1093/bioinformatics/btaf242)
Supplement: btaf242_Supplementary_Data [file btaf242_supplementary_data.pdf]

Supplemental Information

Contents

1 Simulated Data Explanation and Hyperparameter Sweeps **2**
1.1 Multivariate Normal Checkerboard Spatial Anomaly 3
1.2 S-curve Manifold Checkerboard Spatial Anomaly 5
1.3 Spiral Manifold Checkerboard Spatial Anomaly 7
1.4 Multivariate Normal Spiral Shaped Anomaly 9
1.5 Experiments with Joint Gene Expression and Spatial Coordinate Feature Vectors 11

2 Simulating Misalignment **12**
2.1 Simulating Misalignment in Simulated Data 12
2.2 Simulating Misalignment in Visium Data of the Mouse Cerebral Cortex 13

3 Mouse Cortex Stab Wound Analysis Details **14**
3.1 Stab Wound Location 14
3.2 Obtaining aligned ST slices with STalgin 15
3.3 Running *ourMethod* 16
3.4 Running Vespucci 17
3.5 Running MELD 18
3.6 Running STANDs 19
3.7 Known Gene Sets of Interest 20
3.8 GSEA Results 21

4 Spinal Cord Injury Dataset Analysis Details **22**
4.1 Given Data 22
4.2 Running *ourMethod* 22
4.3 Running Vespucci 23
4.4 Running MELD 24
4.5 Full Comparison on all 6 Pairwise Combinations of Condition 24
4.6 Full GSEA results for each method 25
4.7 Visualizing Anomaly Scores Across all Conditions 26

5 Local Two-Sample Testing and Other Methods **27**
5.1 Local Two-Sample Testing 27
5.2 Single-cell methods 29

1 Simulated Data Explanation and Hyperparameter Sweeps

We benchmarked against a method we call *ourMethod* -localManifold and included the results here rather than the main text, which constructs a new data manifold at each spatial anomaly family composed of only the points in that spatial region. We did this to test the hypothesis that the using the entire data manifold, and therefore having access to a more faithful manifold representation, would be beneficial in computing density estimates. For each of the simulated setups we also performed a hyperparameter sweep of *ourMethod* -localManifold.

1.1 Multivariate Normal Checkerboard Spatial Anomaly

We generated two multivariate normal distributions. Both multivariate normal distributions had unit covariance, and for the first distribution, each entry in the mean vector is drawn from a uniform distribution of $(0, 100)$. For the second distribution, each entry in the mean vector is the corresponding entry in the first distribution's mean vector, plus another normal distribution centered at 0.

Condition 1 is created by sampling every spot from the first multivariate normal distribution. Condition 2 is created creating a 3x3 grid of subsquares inside the overall square coordinate grid, and assigning each subsquare to draw from either the first or second distribution based on a checkerboard pattern.

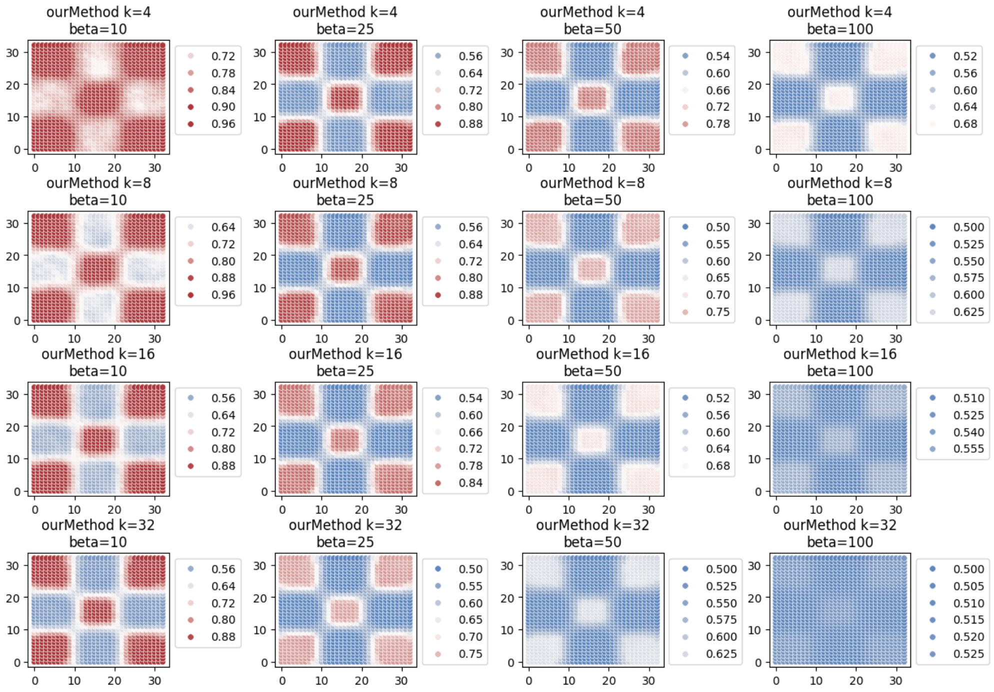

Figure 1: *ourMethod* on a range of hyperparameters. All results shown are for a spatial neighborhood size of 30. All parameters not mentioned were left at default.

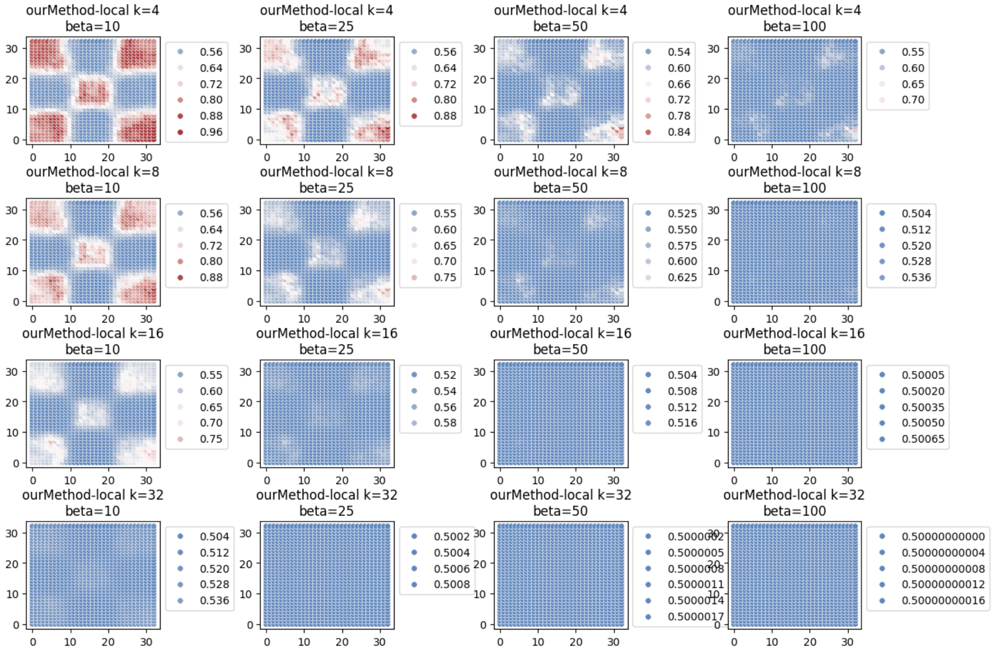

Figure 2: *ourMethod* -localManifold on a range of hyperparameters. All results shown are for a spatial neighborhood size of 30. All parameters not mentioned were left at default.

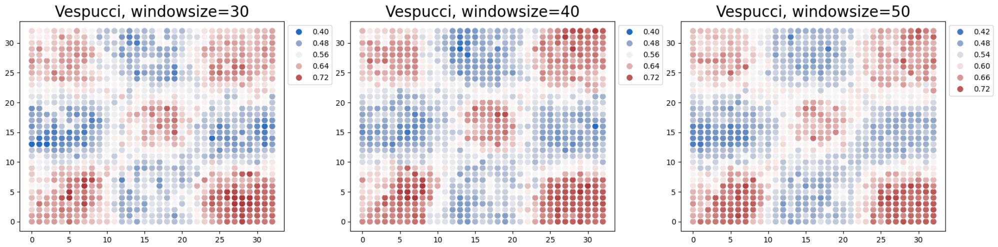

Figure 3: Vespucci with varying window sizes. All other parameters were left at default.

1.2 S-curve Manifold Checkerboard Spatial Anomaly

We generated the manifold data through the make_s_curve function in the sklearn package, and then padding the dimensionaltiy with random noise vectors so that the dimensionaltiy was 100. We then partitioned the manifold into discrete partitions (shown in Figure 2), and assigned subsquares to sample from its assigned discrete region of the manifold. In this way, between the two conditions, some of the corresponding subsquares would draw expression values from the same region of the manifold, and some subsquares would draw from different regions of the manifold between conditions.

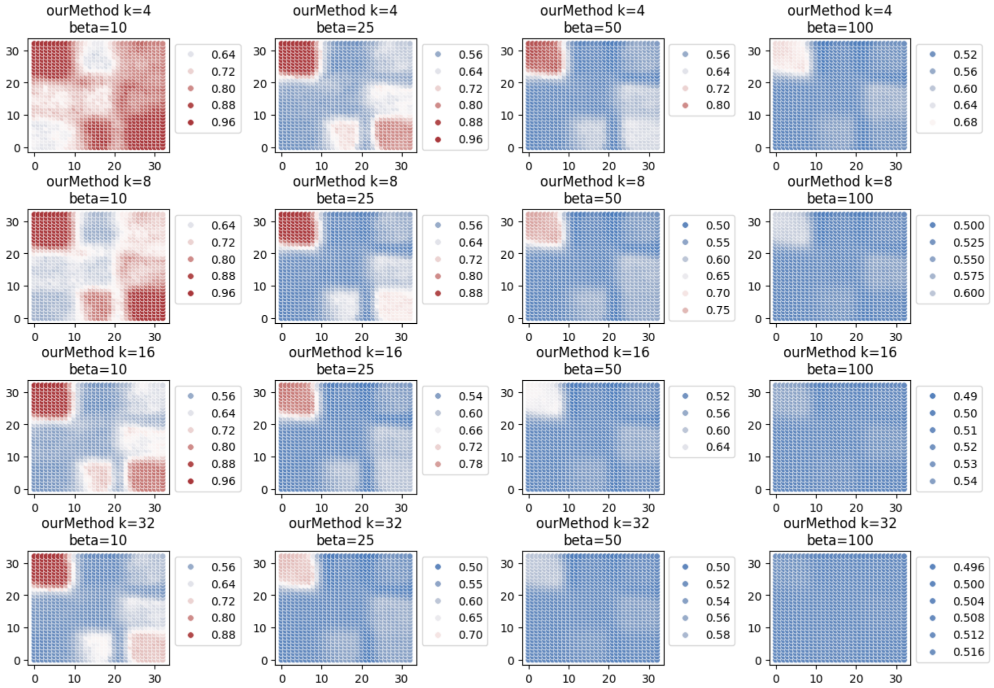

Figure 4: *ourMethod* on a range of hyperparameters. All results shown are for a spatial neighborhood size of 30. All parameters not mentioned were left at default.

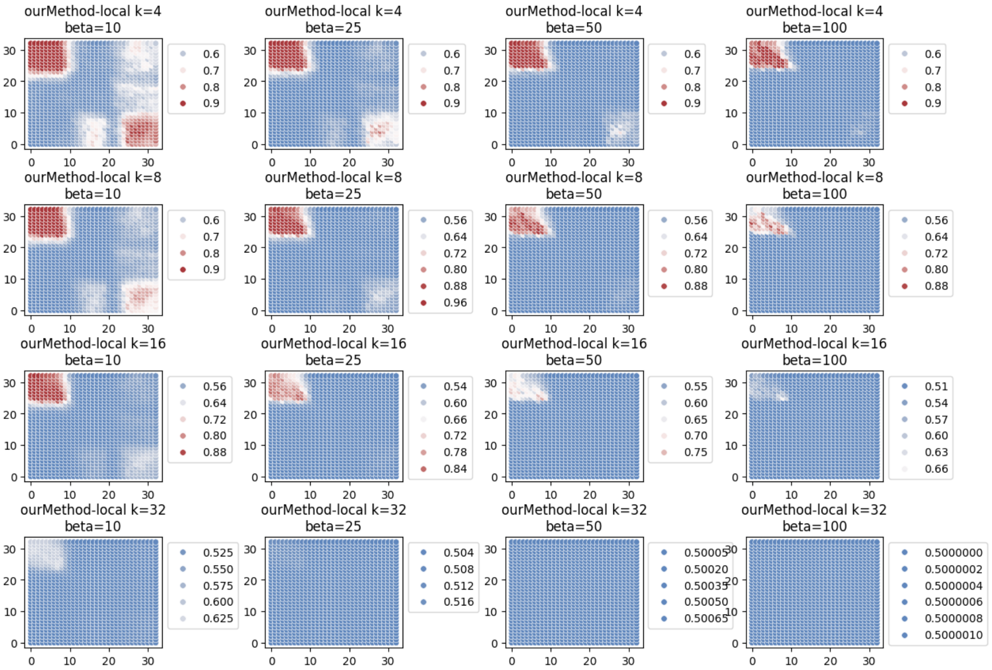

Figure 5: *ourMethod* -localManifold on a range of hyperparameter. All results shown are for a spatial neighborhood size of 30. All parameters not mentioned were left at default.

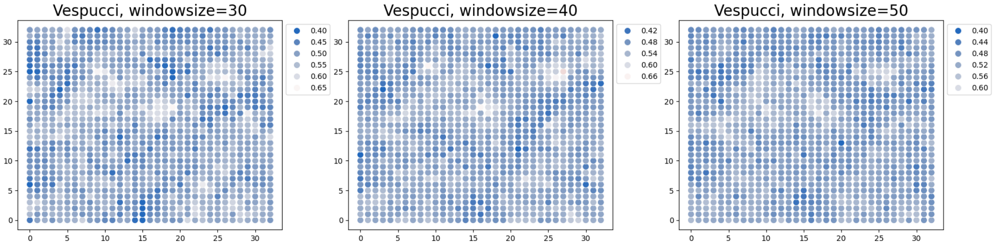

Figure 6: Vespucci with varying window sizes. All other parameters were left at default

1.3 Spiral Manifold Checkerboard Spatial Anomaly

We generated spiral manifold data by embedding a $2D$ spiral manifold structure of 10000 points within 100 dimensions, and then partitioning the manifold space by dividing these 10000 points equally into different regions. We then proceeded identically to the previous simulated example. In this simulation, not only did we use a spiral manifold, where each subsquare sampled it's expression from different parts of the low dimensional manifold, but defined the sampling distribution as over two different regions of the expression manifold for each spot, a proportion p was set for each subsquare Q_i that controlled in what proportion it sampled from 1 or 2 distinct regions. Otherwise, the simulation was set up similarly.

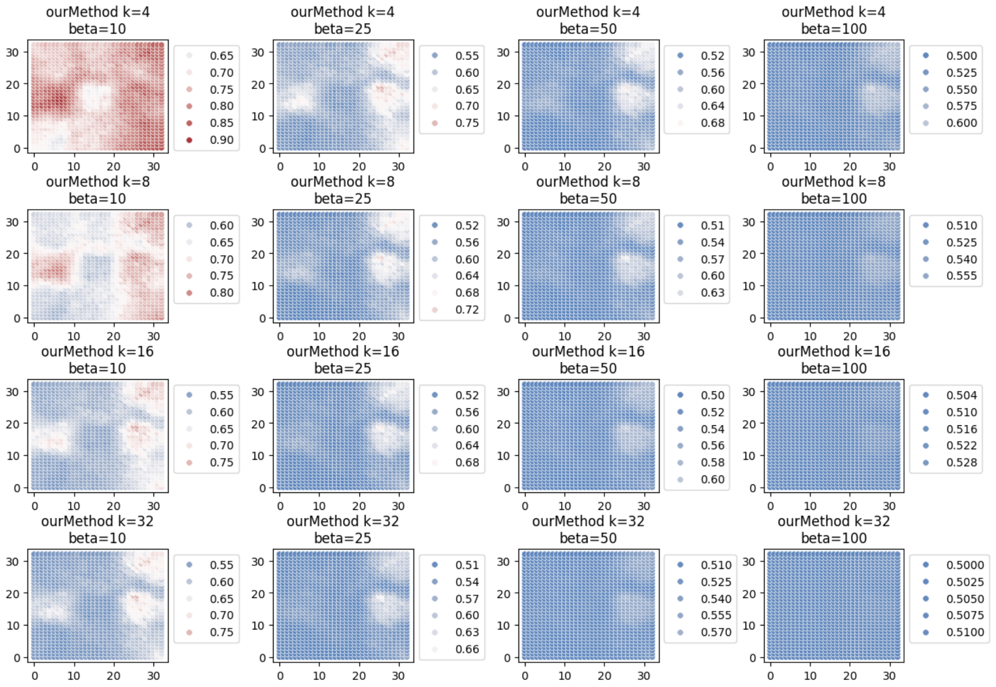

Figure 7: *ourMethod* on a range of hyperparameters All results shown are for a spatial neighborhood size of 30. All parameters not mentioned were left at default.

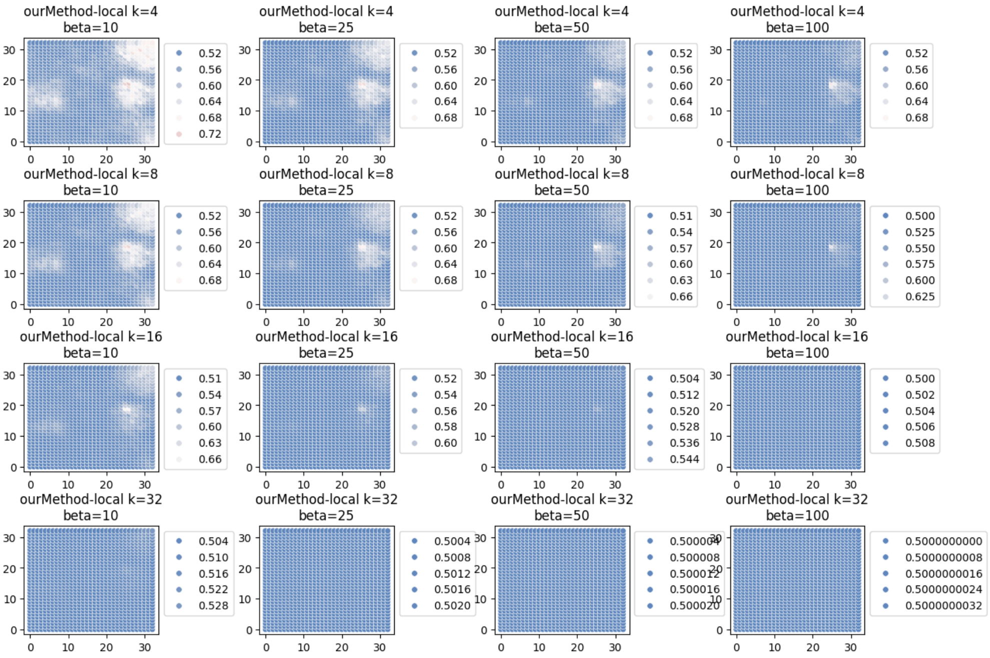

Figure 8: *ourMethod* -localManifold on a range of hyperparameter. All results shown are for a spatial neighborhood size of 30. All parameters not mentioned were left at default.

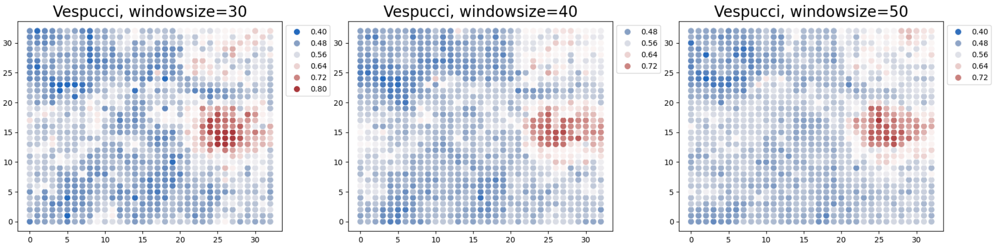

Figure 9: Vespucci with varying window sizes. All other parameters were left at default

1.4 Multivariate Normal Spiral Shaped Anomaly

The expression distribution is the same as what is described in Section 1.1, however, in this case we generate a spiral shape within the coordinate grid, and have all points that lie in this spiral shape be drawn from the two separate multivariate normal distributions, otherwise the spots outside this spiral shape both draw from the first multivariate normal in both conditions. In addition to hyperparameter sweeps of the density estimation hyperparameters, we also performed a hyperparameter sweep of the neighborhood size.

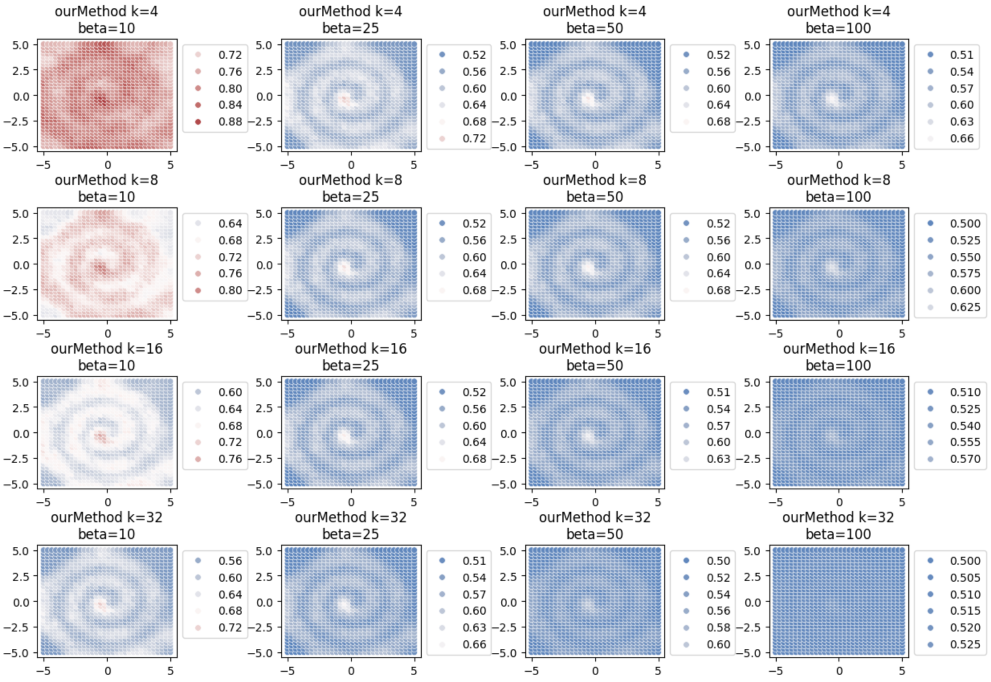

Figure 10: *ourMethod* on a range of hyperparameters. All results shown are for a spatial neighborhood size of 30. All parameters not mentioned were left at default.

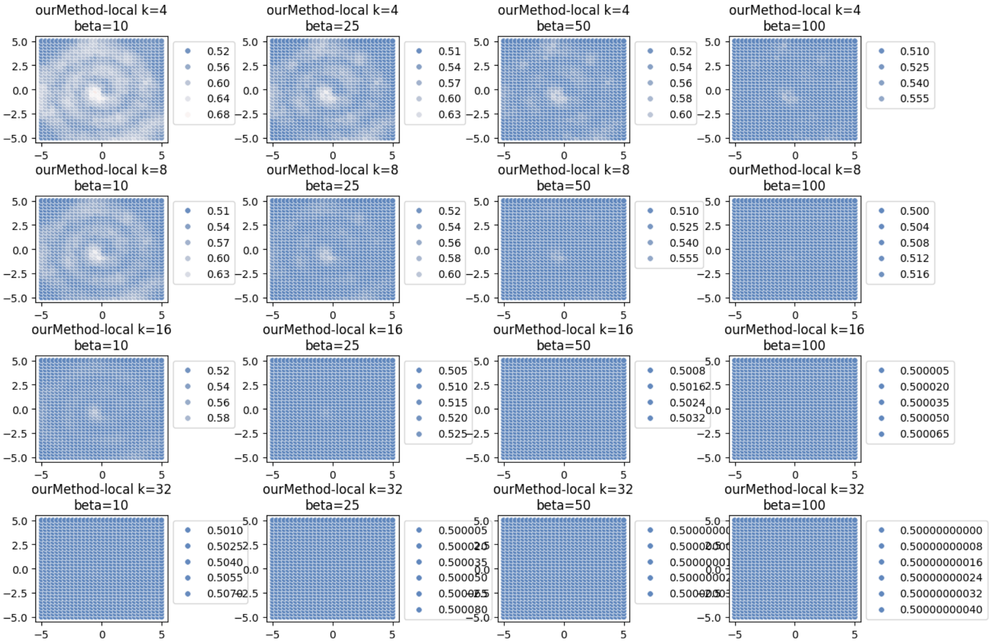

Figure 11: *ourMethod* -localManifold on a range of hyperparameters. All results shown are for a spatial neighborhood size of 30. All parameters not mentioned were left at default.

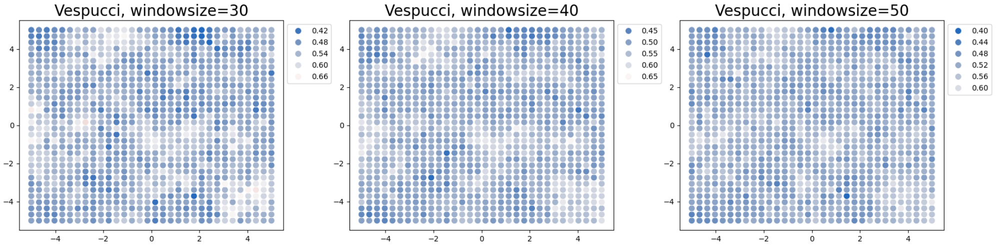

Figure 12: Vespucci with varying window sizes. All other parameters were left at default

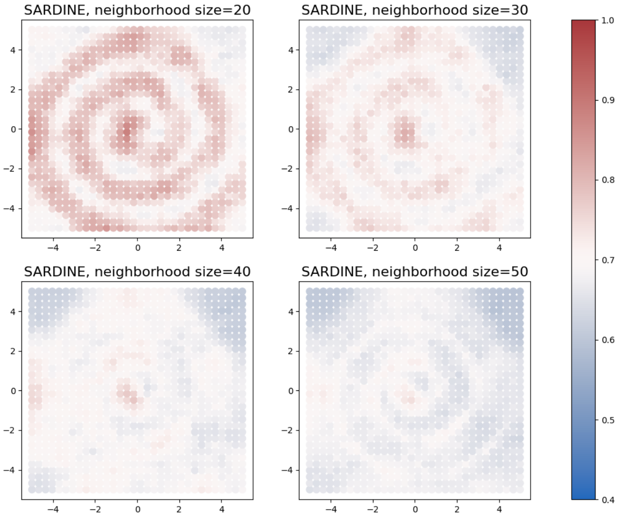

Figure 13: Sardine with varying window sizes

1.5 Experiments with Joint Gene Expression and Spatial Coordinate Feature Vectors

We conducted two simple experiments to demonstrate that existing methods such as MELD and traditional clustering cannot identify spatial anomalies. We hypothesize that even though these existing methods can be adapted to the spatial anomaly detection problem by utilizing concatenated gene expression and spatial coordinate vectors as input to similarity measures, that the performance of these methods is inadequate.

First, we assessed whether running MELD on the similarity graph formed by joint gene expression and spatial coordinate information could lead to spatial anomaly detection at the level or better than Sardine. To assess this, we used the simulation setup shown in Figure $2C$ in the manuscript, corresponding to section 1.2 in the supplement. We ran MELD on the concatenated gene expression and spatial coordinate vectors, and show the output below.

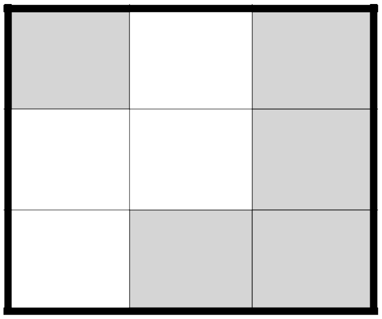

Figure 14: Ground Truth of Spatial Anomaly Pattern

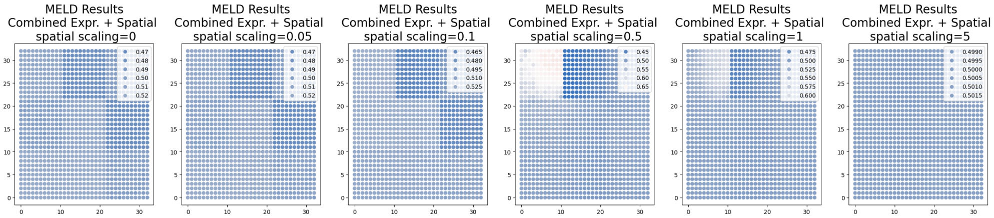

Figure 15: MELD on combined gene expression and spatial coordinate vectors, with different spatial scaling factors

The scaling refers to a multiplicative weight on the spatial coordinates, to either amplify or remove the contribution of spatial distances in the similarity measure. We note that the lack of a canonical weight serious hinders the interpretability and applicability of this approach. Regardless, we found that among all of the scaling weights chosen, none had an AUC comparing to Sardine (max AUC was 0.636, compared to the AUC of 0.98 of Sardine).

We also ran additional experiment to assess whether traditional clustering techniques on the joint gene expression and spatial coordinates would be competitive with Sardine. As before, we tested a variety of scaling factors for the spatial coordinates, which were then used in a traditional PCA, followed by kmeans clustering approach, where $k = 2$. Below shows the output of this approach, where w observe that yet again the accuracy is far lower than Sardine (max AUC = 0.71)

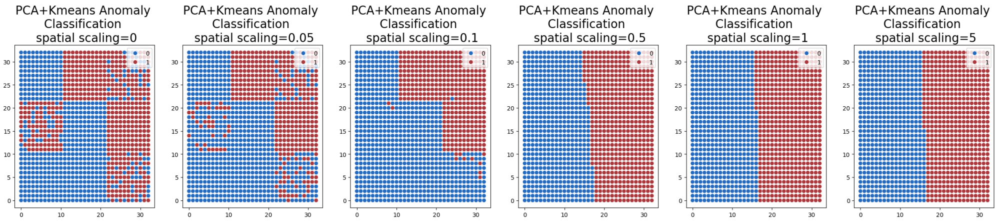

Figure 16: PCA followed by KMeans, k = 2, on combined gene expression and spatial coordinate vectors, with different spatial scaling factors

2 Simulating Misalignment

2.1 Simulating Misalignment in Simulated Data

To test the effects of modest amounts of misalignment, we performed the following experiment. Using the simulated dataset shown in Figure $2B$ in the main manuscript, corresponding to Supplementary section 1.1, we randomly permuted the spots in the following manner:

Given the ground truth datasets for condition 1 and condition 2, we set two user-defined parameters: (1) a neighborhood distance i, and (2) a number of permutations j. For j iterations, we randomly chose a spot within the condition 2 dataset, and swapped its expression features with a neighbor within the i closest spots to itself.

Because the original datasets were perfectly aligned to one another, this process simulates the effect of modest misalignment. We reason that this approximation of misalignment is reasonable because due to geometric constraints in most alignment algorithms, spots that are misaligned will still likely within the general vicinity of their true alignment location. We tried this process with neighborbood distances $= [10, 50, 100, 200]$ and number of permutations $= [100, 200, 400, 800]$

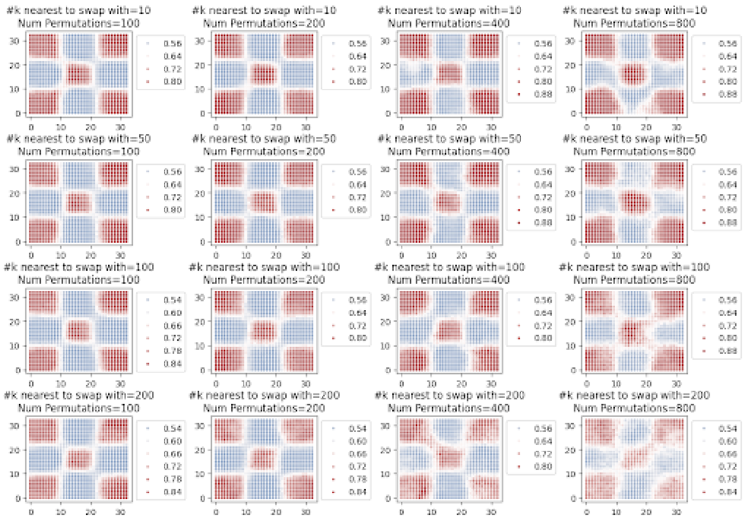

Figure 17: Effect of Simulated Misalignment, varying distance to permute and number of permutations

For each pair of hyperparameters, we ran Sardine with the same parameters as what was shown in Figure $2C$. Afterword, we computed the Pearson correlation of the found anomaly scores with the output of Sardine where the slices are perfectly aligned to one another. We found that the minimum Pearson correlation was ≈ 0.91, thus indicating that our method is robust to modest amounts of misalignment.

2.2 Simulating Misalignment in Visium Data of the Mouse Cerebral Cortex

We also assessed the impact of misalignment on real Visium data. To do so, we repeated the analysis of the Mouse Cortex Cerebral Cortex with Sardine. In the original experiment, the slices were all aligned onto a common coordinate system using the package STalign. To simulate misalignment, we instead only performed centering and rotation of the individual slices to align them onto a common coordinate system.

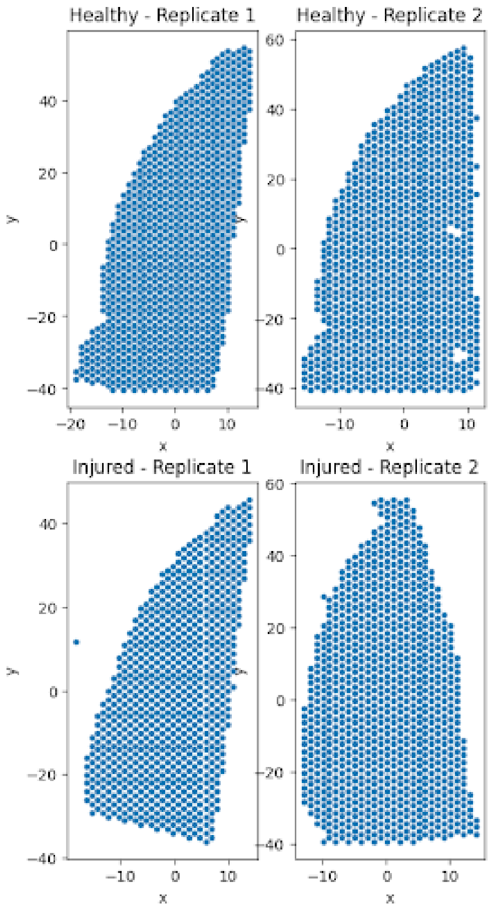

Figure 18: Effect of Simulated Misalignment, varying distance to permute and number of permutations

As before with simulated data, we ran Sardine with the same hyperparameters as in the original analysis, and then computed the Pearson correlation of the anomaly scores from the *misalignment* slices with the ones aligned by STalign. As before, we found that the Pearson correlation between the two was very high (≈ 0.88).

3 Mouse Cortex Stab Wound Analysis Details

3.1 Stab Wound Location

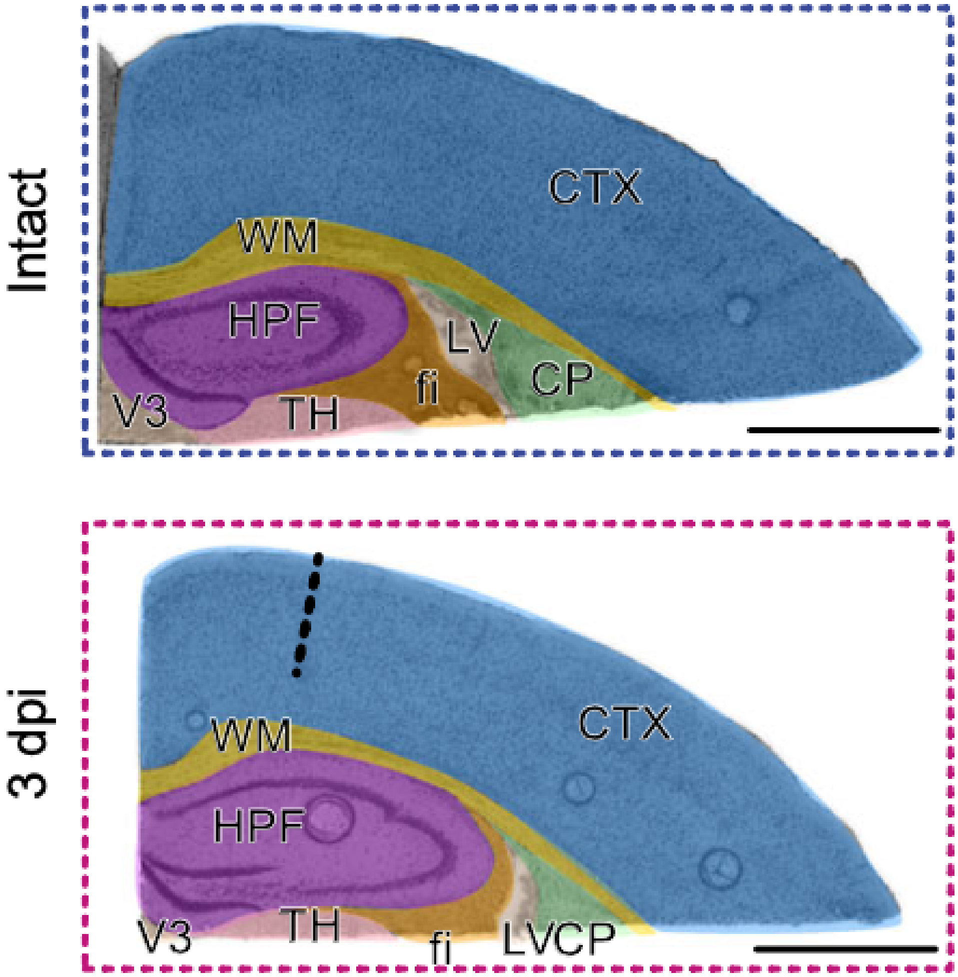

Figure 19: Stab wound location on the injured mice replicates. Image taken directly from the original authors (Koupourtidou *et al.*, 2024)

3.2 Obtaining aligned ST slices with STalgin

In order to place all slices of the mouse cortex stab wound dataset onto a common coordinate system, we ran STalign Clifton *et al.* (2023), a recent cross-slice alignment method that can operate only on the spatial data.

The data provided by Koupourtidou *et al.* (2024) consists of two replicates each for the healthy and post injury conditions. The two replicates for each slice originally came on the same data file, thus we manually divided the coordinate space and corresponding expression data of the spots to split the replicates, resulting in 4 slices. After centering and rotating so that each slice was in the same orientation, we set one of the healthy replicate slices to be the reference slice, and used STalign to align the other 3 slices to it. STalign was run with the following parameters:

```
params = {
          'niter':1000,
          'device':device,
          'diffeo_start':100,
          'a':250,
          'epV':1000,
          'sigmaB':0.1,
          'muB': torch.tensor([0,0,0]), # black is background in target,
          }
```

3.3 Running *ourMethod*

In order to be consistent with Vespucci, we used the same default spatial window size of 50 spots from each condition to create the spatial window. Hyperparameters for the density estimator were chosen based off of the consistent results in simulated data, with $\beta = 25, k = 8$, and all other parameters being default for MELD.

3.4 Running Vespucci

The resulting expression data and spatial coordinate after aligning with STalgin were passed into Teo *et al.* (2024) to compute scores for each spot corresponding the expression perturbation.

Vespucci was run with entirely default parameters, passing in only the expression matrix and a metadata dataframe that contained a barcode ID, X and Y spatial coordinates, replicate ID, and condition label for each spot.

Vespucci is composed of two distinct sections: a method to calculate spatial AUCs (what Vespucci uses to score how anomalous a particular spot is), and then a differential expression procedure that takes as input these spatial AUCs. Due to a found bug in the differential expression portion of their code, we only ran the spatial AUC method to compare against *ourMethod* .

3.5 Running MELD

We ran MELD with $\beta = 60$, which is the recommended parameter value in the original publication, as well as $k = 8$. All other parameters were left as default.

3.6 Running STANDs

Because STANDs can make use of histology images, we did not the version of the mouse cortex data that was aligned using STalign. Instead, we passed the original slices, along with the histoly images, into STANDs. STANDs requires pretraining and fitting on the reference dataset, and then evaluating on the perturbed condition dataset.

The data was read in with patch size=16 and $ngenes = 3000$. Following the tutorial, we ran the fitting procedure with patch size=16, number of epochs = 20, and batch size = 64, with all other parameters being default. For the anomaly detection, we ran with number of epochs = 10 and batch size = 64.

3.7 Known Gene Sets of Interest

We used the following preset gene sets for marker genes of interest. These gene lists consist of genes mentioned in (Koupourtidou *et al.*, 2024):

1. Reactive Astrocyte Marker Genes: *Gfap, Lcn2, Serpina3n, Vim, Lgals1, Fabp7, Tspo*
2. Microglia Marker Genes: *Aif1, Csf1r, Cd68, Tspo*
3. Type 1 Interferon Pathway Genes: *Ifitm3, Ifit3, Bst2, Isg15, Ifit3b, Irf7, Ifit1, Ifi27l2a, Oasl2, Oas1a*

3.8 GSEA Results

We upload two sources of the full results of the GSEA analysis. First, we attached urls to the results of running the GSEA analysis using webGestalt on the anomaly scores of each method:

1. *ourMethod* : `https://www.webgestalt.org/results/1743118169/#`
2. STANDS: `https://2024.webgestalt.org/results/1737569257/#`
3. MELD: No significant results returned by webGestalt
4. Vespucci: `https://2024.webgestalt.org/results/1737569712/#`

In case the above links no longer work, we also include downloaded results from the above analysis stored in our github repository.

4 Spinal Cord Injury Dataset Analysis Details

4.1 Given Data

Unlike the mouse cortex stab wound dataset, this spinal cord injury dataset came prealigned. Thus, we did not perform any additional spatial registration and left the coordinates as they were given. Unfortunately, due to the prealignment, as well as the fact that different replicates were imaged together in the histology slice, we unable to run methods that require histology images such as STANDs

4.2 Running *ourMethod*

In order to be consistent with Vespucci, we used the same default spatial window size of 50 spots from each condition to create the spatial window. Hyperparameters for the density estimator were chosen based off of the consistent results in simulated data, with $\beta = 15, knnk = 8$, and all other parameters being default for MELD.

4.3 Running Vespucci

Vespucci was run with default parameters on every run.

4.4 Running MELD

We ran MELD with $\beta = 60$, which is the recommended parameter value in the original publication, as well as $knnk = 8$. All other parameters were left as default.

4.5 Full Comparison on all 6 Pairwise Combinations of Condition

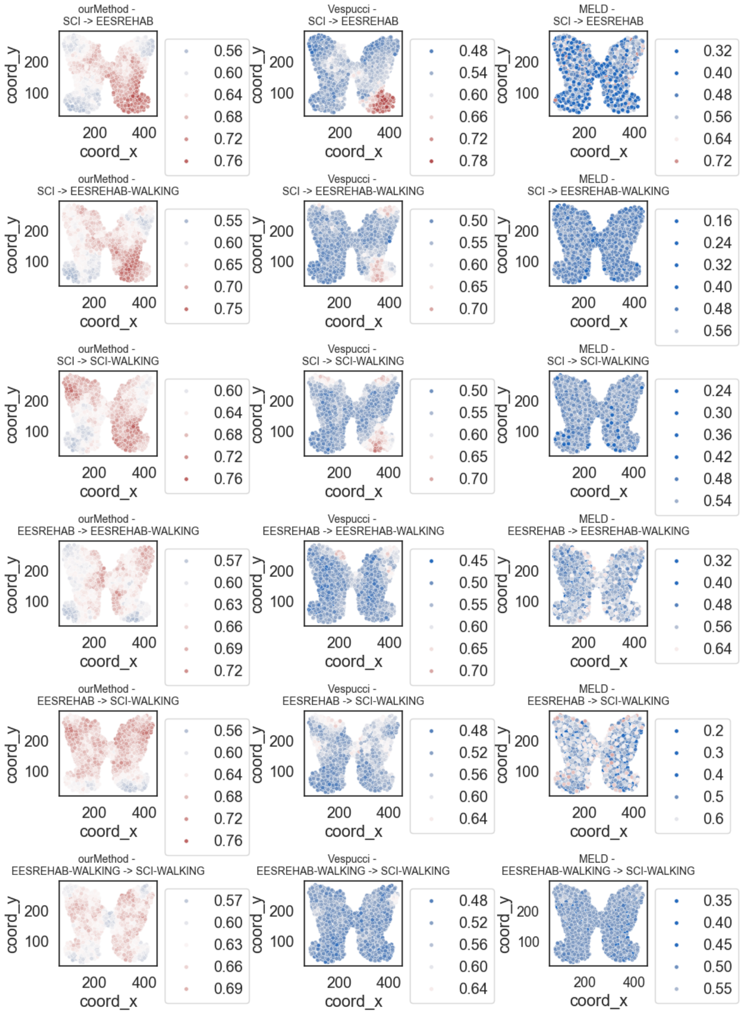

Figure 20: A comparison of Sardine, MELD, and Vespucci across all 6 pairs of conditions in the spinal cord injury dataset

4.6 Full GSEA results for each method

We upload two sources of the full results of the GSEA analysis. First, we attached urls to the results of running the GSEA analysis using webGestalt on the anomaly scores of each method:

1. *ourMethod* : `https://2024.webgestalt.org/results/1737570684/#`
2. MELD: No significant results returned by webGestalt
3. Vespucci: `https://2024.webgestalt.org/results/1737570335/#`

In case the above links no longer work, we also include downloaded results from the above analysis stored in our github repository.

4.7 Visualizing Anomaly Scores Across all Conditions

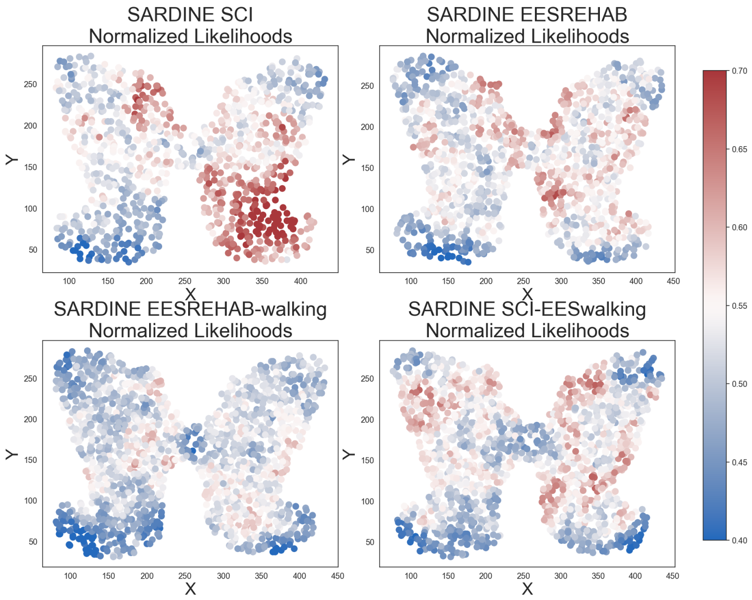

Figure 21: Spinal Cord Injury anomaly scores reported by Sardine, where each condition's average density at each spot is normalized by the sum of the average densities from all 4 conditions.

5 Local Two-Sample Testing and Other Methods

5.1 Local Two-Sample Testing

Here we present the framework for local two-sample testing following Landa *et al.* (2020). Let f_0, f_1 be probability density functions supported on a measurable space $\mathcal{X}$. For *class prior* $p \in (0, 1)$, let X be a random variable generated by sampling from f_1 with probability p, and from f_0 with probability $1-p$. Class assignment is modeled with random variable $Z \sim \text{Bernoulli}(p)$, and one has

$$(X|Z=1) \sim f_1, \quad (X|Z=0) \sim f_0.$$

We then take $(\boldsymbol{x}_1, z_1), \ldots, (\boldsymbol{x}_n, z_n)$ as i.i.d. samples from the joint distribution of X and Z, where $\boldsymbol{x}_i \in \mathcal{X}$ and $z_i \in \{0, 1\}$. The class values z_i partition the $\boldsymbol{x}$-samples into $\boldsymbol{X} = \{\boldsymbol{x}_i : z_i = 0\}$ and $\boldsymbol{X'} = \{\boldsymbol{x}_i : z_i = 1\}$. One may equivalently view the above setup as: we are given datasets $\boldsymbol{X}$ and $\boldsymbol{X'}$, respectively i.i.d. samples from f_0 and f_1. Each sample comes with an implicit class label, and we can estimate p by the fraction of the dataset that belongs to class 1. In applications relevant to our work, one can imagine $\boldsymbol{X}$ and $\boldsymbol{X'}$ as single-cell RNA-seq datasets coming from different but comparable biological conditions, and with the same preprocessing applied to each dataset.

Given sample $\boldsymbol{x} \in \mathcal{X}$, define the class posterior

$$\begin{aligned} m(\boldsymbol{x}) &:= \mathbb{E}(Z|X=\boldsymbol{x}) \\ &= \mathbb{P}(Z=1|X=\boldsymbol{x}), \end{aligned} \tag{1}$$

so that by Bayes' theorem,

$$\begin{aligned} m(\boldsymbol{x}) &= \frac{pf_1(\boldsymbol{x})}{pf_1(\boldsymbol{x}) + (1-p)f_0(\boldsymbol{x})} \\ &= \frac{pf_1(\boldsymbol{x})}{f(\boldsymbol{x})}\,, \end{aligned} \tag{2}$$

where $f(\boldsymbol{x}) := (1-p)f_0(\boldsymbol{x}) + pf_1(\boldsymbol{x})$.

In two-sample testing, one attempts to reject the null hypothesis $f_1(\boldsymbol{x}) = f_0(\boldsymbol{x})$ for all $\boldsymbol{x} \in \mathcal{X}$. In *local* two-sample testing, one determines whether $f_1(\boldsymbol{x}) > f_0(\boldsymbol{x})$ for all $\boldsymbol{x} \in R$, for possibly many regions $R \subset \mathcal{X}$. Each region R thus yields an informative alternative hypothesis: one can identify *where* the densities differ in addition to rejecting the null. Prior to Landa et al., Kim *et al.* (2019) proposed to use local statistics of the form $(m(\boldsymbol{x}) - p)^2$, allowing one to reject the global null $f_0 = f_1$ and accept a pointwise alternative of the form $f_0(\boldsymbol{x}) \neq f_1(\boldsymbol{x})$ for some $\boldsymbol{x} \in \mathcal{X}$. Regions R used by Kim *et al.* (2019) thus consist of single points $\{\boldsymbol{x}\}$ for $\boldsymbol{x} \in \mathcal{X}$. This approach is limited: we cannot conclude that $f_1(\boldsymbol{x}_i) \neq f_0(\boldsymbol{x}_i)$ with finite-sample probabilistic guarantees, unless f_0 and f_1 are sufficiently smooth.

Guarantees at a given $\boldsymbol{x} \in \mathcal{X}$ thus depend on smoothness of f_0, f_1, plus an abundance of nearby class labels. This reasoning suggests that statistics used in Kim *et al.* (2019) are too local, and should instead be defined in terms of small neighborhoods of data points. Following this reasoning, Landa *et al.* (2020) put forward the idea that regions of interest can be represented by discrete probability measures supported on the observed data. In place of a collection of regions $\mathcal{R} = \{R_j\}_{j=1}^N$, we consider a collection of probability vectors $\mathcal{F} = \{\boldsymbol{w}_j\}_{j=1}^N$ supported on the observed data. Thus, the null hypothesis $f_0(\boldsymbol{x}) = f_1(\boldsymbol{x})$ can be rejected for a local alternative in the form of one of the $\boldsymbol{w}_j$, if for instance

$$\sum_{i=1}^{n} \boldsymbol{w}_j(\boldsymbol{x}_i)(f_1(\boldsymbol{x}_i) - f_0(\boldsymbol{x}_i)) > 0\,. \tag{3}$$

Note that the Landa *et al.* (2020) formulation subsumes the region-based formulation: any collection of regions $\mathcal{R} = \{R_j\}_{j=1}^N$ can be encoded as a set of weight vectors $\mathcal{F} = \{\boldsymbol{w}_j\}_{j=1}^N$ by defining $\boldsymbol{w}_j$ as the uniform probability vector on region R_j.

In place of the density difference (3), Landa *et al.* (2020) use the following discrepancy score, using (2) in the third line below:

$$\begin{aligned} s_{\text{Landa}}(\boldsymbol{x}) &:= m(\boldsymbol{x}) - p \\ &= \mathbb{P}(Z = 1 | X = \boldsymbol{x}) - p \\ &= \frac{p f_1(\boldsymbol{x})}{f(\boldsymbol{x})} - p \\ &= p(1-p) \left(\frac{f_1(\boldsymbol{x}) - f_0(\boldsymbol{x})}{f(\boldsymbol{x})} \right) . \end{aligned} \tag{4}$$

We write $\boldsymbol{s}$ for the vector of such scores, as indexed by the samples $\{\boldsymbol{x}_i\}_{i=1}^n$: this is to say $\boldsymbol{s} \in \mathbb{R}^n$, and that $[\boldsymbol{s}]_i = s(\boldsymbol{x}_i)$ for each $\boldsymbol{x}_i$. For $\mathcal{G} \subset \mathcal{F}$, Landa *et al.* (2020) define the parameterized null hypothesis $H_0(\mathcal{G})$ as

$$H_0(\mathcal{G}) : \quad \langle \boldsymbol{w}, \boldsymbol{s} \rangle \leq 0, \quad \forall \boldsymbol{w} \in \mathcal{G} \,,$$

denoting $H_0(\mathcal{F})$ by H_0. For $\boldsymbol{w} \in \mathcal{F}$ and $\gamma \geq 0$, define $H_1(\boldsymbol{w}, \gamma)$ as a specific alternative to $H_0(\boldsymbol{w})$ via

$$H_1(\boldsymbol{w}, \gamma) : \quad \langle \boldsymbol{w}, \boldsymbol{s} \rangle > \gamma \|\boldsymbol{w}\|_2 \,.$$

While vector $\boldsymbol{s}$ is defined in terms of the true density difference (4) at each datapoint, Landa *et al.* (2020) use the vector of class labels $\boldsymbol{z} = (z_i)_{i=1}^n$ in the inner product $\langle \boldsymbol{w}, \boldsymbol{z} - p \rangle$ as an unbiased estimator of $\langle \boldsymbol{w}, \boldsymbol{s} \rangle$, ultimately using the statistic

$$\mathcal{S}_{\text{Landa}}(\boldsymbol{w}) := \frac{\langle \boldsymbol{w}, \boldsymbol{z} - p \rangle}{\|\boldsymbol{w}\|_2},$$

which coincides with the *positive elevated-mean statistic* when $\boldsymbol{w}$ is uniform on a subset of the data.

As an important special case of their framework suited to high-dimensional data, Landa *et al.* (2020) propose to use random walk neighborhoods on the data as weight vectors. Concretely, let $G^{(k)}$ be the k-NN graph built from the data $\boldsymbol{x}_1, \ldots, \boldsymbol{x}_n$, and suppose that each data point $\boldsymbol{x}_i$ is equipped with a collection of "judiciously" chosen time steps, $1 = t_1(i) < t_2(i) < \cdots < t_{M_i}(i)$. Writing $\boldsymbol{w}_{i,j}$ for the distribution induced over the data by a $t_j(i)$-step simple random walk on $G^{(k)}$ started at $\boldsymbol{x}_i$, Landa *et al.* (2020) suggest using the collection of weight vectors $\mathcal{F} = \{\boldsymbol{w}_{i,j}\}$. This approach is especially suited to high-dimensional data adhering to the manifold hypothesis: regions of interest are defined on the data directly, with Landa *et al.* (2020) arguing: *"in such cases it is inappropriate to define regions of interest according to the high-dimensional ambient space, as the underlying notion of locality can be fundamentally different."*

5.2 Single-cell methods

Below, we describe how each method operates on data $\boldsymbol{X}, \boldsymbol{X}'$ drawn from two biological conditions within the above framework. We denote $n_0 := |\boldsymbol{X}|$ and $n_1 := |\boldsymbol{X}'|$, and $n := n_0 + n_1$.

MELD, Burkhardt *et al.* (2021) Given integer $k > 0$, let $G^{(k)}$ denote the symmetrized (undirected) k-NN graph formed from $\boldsymbol{X} \cup \boldsymbol{X}'$. We suppose $G^{(k)}$ is weighted, with edge weights w_e for $e = \{u, v\}$ some function of the endpoints $\boldsymbol{x}_u$ and $\boldsymbol{x}_v$. Let $\boldsymbol{W}$ denote weighted adjacency matrix of $G^{(k)}$, and define degree matrix $\boldsymbol{D}$ as the diagonal matrix with entries $\boldsymbol{D}_{uu} = \sum_v \boldsymbol{W}_{uv}$. The weighted graph Laplacian $\boldsymbol{L}$ is

$$\boldsymbol{L} = \boldsymbol{D} - \boldsymbol{W} \tag{5}$$

As matrix $\boldsymbol{L}$ is symmetric, it admits eigendecomposition $\boldsymbol{L} = \boldsymbol{\Psi}\boldsymbol{\Lambda}\boldsymbol{\Psi}^{-1}$, where $\boldsymbol{\Lambda}$ denotes an ordered set of Laplacian eigenvalues, $\boldsymbol{\Lambda} := \{0 = \lambda_1 \leq \lambda_2 \leq \cdots \leq \lambda_n\}$ or the diagonal matrix with these eigenvalues along the diagonal, depending on context. Likewise, $\boldsymbol{\Psi}$ is either the set of column eigenvectors $\{\boldsymbol{\psi}_i\}_{i=1}^n$, or the $n \times n$ matrix $[\boldsymbol{\psi}_1\, \boldsymbol{\psi}_2\, \ldots\, \boldsymbol{\psi}_n]$ with eigenvectors as columns, constituting an orthonormal basis for $\mathbb{R}^n$.

We use *graph Fourier basis* and *graph Laplacian eigenvectors* interchangeably. The *graph Fourier transform* $\hat{\boldsymbol{f}} \in \mathbb{R}^n$ of signal $\boldsymbol{f} \in \mathbb{R}^n$ has ℓ-th coordinate (i.e. Fourier coefficient corresponding to frequency $\equiv$ eigenvalue λ_ℓ) given by

$$\hat{\boldsymbol{f}}_\ell = \sum_i \boldsymbol{f}(i)\boldsymbol{\psi}_\ell(i) = \langle \boldsymbol{f}, \boldsymbol{\psi}_\ell \rangle.$$

Thus, $\hat{\boldsymbol{f}}$ may be expressed as the matrix-vector product

$$\hat{\boldsymbol{f}} = \boldsymbol{\Psi}^T \boldsymbol{f},$$

The *inverse graph Fourier transform* is $\check{\boldsymbol{g}} := \boldsymbol{\Psi}\boldsymbol{g}$. To smooth the condition labels $\boldsymbol{m}$ into density estimates $\boldsymbol{d}$, Burkhardt *et al.* (2021) introduce the *manifold heat filter*:

$$\boldsymbol{d}^\star = \arg\min_{\boldsymbol{z}} \|\boldsymbol{m} - \boldsymbol{d}\|_2^2 + \beta \boldsymbol{d}^T \boldsymbol{J} \boldsymbol{d}, \tag{6}$$

where

$$\boldsymbol{J} = \exp(\beta(\boldsymbol{L}/\lambda_{\max} - \alpha \boldsymbol{I})^\rho) - \boldsymbol{I}\,.$$

Let

$$\boldsymbol{H} = \exp(-\beta(\boldsymbol{L}/\lambda_{\max} - \alpha \boldsymbol{I})^\rho)\,,$$

so that $\boldsymbol{d}^\star$ in (6) can be recovered as $\boldsymbol{H}\boldsymbol{m}$, or equivalently as

$$\boldsymbol{d}^\star = \boldsymbol{\Psi} h(\Lambda) \boldsymbol{\Psi}^T\,, \quad h(\lambda) := \exp(-\beta(\lambda/\lambda_{\max} - \alpha)^\rho)\,.$$

DA-seq, Zhao *et al.* (2021) The condition discrepancy used by Zhao *et al.* (2021) is, for $\boldsymbol{x} \in \mathcal{X}$

$$\begin{aligned} d_{\text{DA-seq}}(\boldsymbol{x}) &= \frac{f_1(\boldsymbol{x}) - f_0(\boldsymbol{x})}{f_1(\boldsymbol{x}) + f_1(\boldsymbol{x})} \\ &= \frac{P(Z=1|X=\boldsymbol{x})/p - P(Z=0|X=\boldsymbol{x})/(1-p)}{P(Z=1|X=\boldsymbol{x})/p + P(Z=0|X=\boldsymbol{x})/(1-p)} \\ &= \frac{m(\boldsymbol{x})/p - (1-m(\boldsymbol{x}))/(1-p)}{m(\boldsymbol{x})/p + (1-m(\boldsymbol{x}))/(1-p)}\,, \end{aligned}$$

where the terms $m(\boldsymbol{x})/p$ and $(1 - m(\boldsymbol{x}))/(1-p)$ are estimated as follows. Let $G^{(k)}$ denote the k-NN graph associated to the data $\boldsymbol{X} \cup \boldsymbol{X}'$. Let $N_1(\boldsymbol{x}_i; k)$ and $N_0(\boldsymbol{x}_i; k)$ be the number of cells among the k nearest

neighbors of $\boldsymbol{x}_i$ that are in condition 1 or 0, respectively. Let $\mathcal{F}_k = \{\boldsymbol{w}_{i,k}\}_{i=1}^n$, where weight vector $\boldsymbol{w}_{i,k}$ is the uniform measure on the k nearest neighbors of $\boldsymbol{x}_i$. Defining

$$g_1(\boldsymbol{x}_i; k) := \frac{N_1(\boldsymbol{x}_i; k)/k}{n_1/n}, \quad g_0(\boldsymbol{x}_i; k) := \frac{N_0(\boldsymbol{x}_i; k)/k}{1 - n_1/n},$$

one equivalently has

$$g_1(\boldsymbol{x}_i; k) = \frac{\langle \boldsymbol{w}_{i,k}, \boldsymbol{z} \rangle}{n_1/n}, \quad g_0(\boldsymbol{x}_i; k) = \frac{\langle \boldsymbol{w}_{i,k}, 1 - \boldsymbol{z} \rangle}{1 - n_1/n},$$

where $\langle \boldsymbol{w}_{i,k}, \boldsymbol{z} \rangle$ and $\langle \boldsymbol{w}_{i,k}, 1 - \boldsymbol{z} \rangle$ are estimators for the complementary posterior probabilities $m(\boldsymbol{x})$ and $1 - m(\boldsymbol{x})$ given by the standard k-NN classifier. The statistic used by DA-seq associated to weight vector $\boldsymbol{w}_{i,k}$ is:

$$\mathcal{S}(\boldsymbol{w}_{i,k}; k) := \frac{g_1(\boldsymbol{x}_i; k) - g_0(\boldsymbol{x}_i; k)}{g_1(\boldsymbol{x}_i; k) + g_0(\boldsymbol{x}_i; k)}. \tag{7}$$

Instead of using a single k, Zhao *et al.* (2021) use a sequence of nearest neighborhood scales, $\boldsymbol{k} = (k_1, \ldots, k_\ell)$. Let $\boldsymbol{\mathcal{S}}_i$ denote the *vector* of the statistics $\mathcal{S}(\boldsymbol{w}_{i,k}; k)$ across these scales,

$$\boldsymbol{\mathcal{S}}_i = (\mathcal{S}(\boldsymbol{w}_{i,k}; k_1), \ldots, \mathcal{S}(\boldsymbol{w}_{i,k}; k_\ell)).$$

By training a regularized logistic classifier, Zhao *et al.* (2021) yield an estimator $\hat{m}_i$ of $m(\boldsymbol{x}_i)$ using an optimal convex combination of the statistics $\boldsymbol{\mathcal{S}}_i$. This in turn yields an estimator for the condition discrepancy $d_{\text{DA-seq}}(\boldsymbol{x}_i)$ at $\boldsymbol{x}_i$, given by

$$\tilde{d}_i = \frac{\tilde{m}_i/p - (1 - \tilde{m}_i)/(1-p)}{\tilde{m}_i/p + (1 - \tilde{m}_i)/(1-p)}.$$

These estimators are used further for clustering and differential expression analysis.

Milo, Dann *et al.* (2022) For a given $k \geq 1$, let $G^{(k)}$ denote the symmetrized k-NN graph for the data $\boldsymbol{X} \cup \boldsymbol{X}'$. To construct a collection of neighborhoods, Dann *et al.* (2022) first subsample the data $\{\boldsymbol{x}_i\}_{i=1}^n$, with the default by a factor of ten, and we lose no generality supposing that $\{\boldsymbol{x}_i\}_{i=1}^s$ for $s < n$ are these subsampled cells.

For each $\boldsymbol{x}_i$, $1 \leq i \leq s$, the mean $\bar{\boldsymbol{x}}_i$ of the k nearest neighbors of $\boldsymbol{x}_i$ is computed (alternately, coordinate-wise median can be used). Let $[\boldsymbol{x}_i]$ denote the nearest $\boldsymbol{x} \in \boldsymbol{X} \cup \boldsymbol{X}'$ to $\bar{\boldsymbol{x}}_i$. The set of all such $[\boldsymbol{x}_i]$ for $1 \leq i \leq s$ corresponds to a subset of indices $\mathcal{I} \subset [n]$. Here, $|\mathcal{I}|$ is possibly strictly less than s, but $\mathcal{I}$ may not be contained in $[s]$. After these neighborhoods have been defined, differential abundance testing is performed using edgeR.

References

Burkhardt, D. B. *et al.* (2021). Quantifying the effect of experimental perturbations at single-cell resolution. *Nature biotechnology*, **39**(5), 619–629.

Clifton, K. *et al.* (2023). Stalign: Alignment of spatial transcriptomics data using diffeomorphic metric mapping. *Nature communications*, **14**(1), 8123.

Dann, E. *et al.* (2022). Differential abundance testing on single-cell data using k-nearest neighbor graphs. *Nature biotechnology*, **40**(2), 245–253.

Kim, I. *et al.* (2019). Global and local two-sample tests via regression.

Koupourtidou, C. *et al.* (2024). Shared inflammatory glial cell signature after stab wound injury, revealed by spatial, temporal, and cell-type-specific profiling of the murine cerebral cortex. *Nature Communications*, **15**(1), 2866.

Landa, B. *et al.* (2020). Local two-sample testing over graphs and point-clouds by random-walk distributions. *arXiv preprint arXiv:2011.03418*.

Teo, A. Y. Y. *et al.* (2024). Identification of perturbation-responsive regions and genes in comparative spatial transcriptomics atlases. *bioRxiv*, pages 2024–06.

Zhao, J. *et al.* (2021). Detection of differentially abundant cell subpopulations in scrna-seq data. *Proceedings of the National Academy of Sciences*, **118**(22), e2100293118.
